# Effects of deep brain stimulation on functional autonomy and quality of life in Parkinson's disease: A systematic review and meta-analysis

**DOI:** 10.1177/1877718X261461967

**Published:** 2026-09-21

**Authors:** Laura Culicetto, Maria Cristina De Cola, Fabiana Pia Vitiello, Giulia Marafioti, Viviana Lo Buono, Anita Maria Sophia Cusumano, Chiara Sorbera, Giuseppe Di Lorenzo, Angelo Quartarone, Silvia Marino

**Affiliations:** 1 120349 IRCCS Centro Neurolesi “Bonino-Pulejo”, Messina, Italy; 2 Dipartimento di Scienze Psicologiche, Pedagogiche, Dell’esercizio Fisico e Della Formazione, 18998 Università degli Studi di Palermo, Palermo, Italy

**Keywords:** Parkinson's disease, deep brain stimulation, subthalamic nucleus, autonomy, quality of life, activities of daily living, meta-analysis

## Abstract

**Background:**

Deep brain stimulation (DBS) of the subthalamic nucleus (STN) is an established therapy for advanced Parkinson's disease (PD), primarily targeting motor complications refractory to pharmacological treatment. However, its long-term impact on functional autonomy and quality of life (QoL) remains debated.

**Objective:**

This systematic review with meta-analysis aimed to evaluate the effects of STN-DBS on autonomy and QoL in people with PD.

**Methods:**

Following PRISMA guidelines, a systematic search of PubMed, Scopus, Embase and Web of Science was conducted to identify studies including adults with PD treated with STN-DBS and reporting measures of autonomy and/or quality of life.

**Results:**

Thirty-two studies met inclusion criteria. Meta-analyses showed effect estimates favoring DBS at 6 months (SMD = 0.83[0.38;1.29]) and at 1 year (SMD = 0.49[-0.02;0.99]) for autonomy. Similar trends were observed for QoL, with SMD = 0.60[0.49;0.71] at 6 months and SMD = 0.48[0.0;0.96] at 1 year.

At 2 years, small but significant pooled effects persisted, for both autonomy SMD = 0.40[0.13;0.67] and QoL SMD = 0.25[-0.04;0.53]. However, at 5 years, pooled effects were no longer statistically significant for either autonomy SMD = 0.37[-0.55;1.29] or QoL SMD = −0.01[-0.25;0.22], indicating a progressive attenuation of benefit over time.

**Conclusions:**

STN-DBS provides robust short- and mid-term benefits in functional autonomy and QoL in PD. Long-term outcomes are variable and influenced by disease progression and non-motor symptoms. Standardized outcome reporting, larger multicenter trials, and integration of rehabilitative and psychosocial interventions are needed to optimize benefits.

## Plain language summary

Does Deep Brain Stimulation Help People with Parkinson's Disease Stay Independent and Improve Their Quality of Life?

Parkinson's disease is a long-term condition that affects movement, balance, and many other aspects of daily life. Over time, people with Parkinson's may struggle with everyday activities such as dressing, walking, cooking, or managing household tasks. These difficulties can reduce independence and quality of life.

Deep brain stimulation (DBS) is a surgical treatment used for people whose symptoms are no longer well controlled by medication. It involves placing small electrodes in a specific area of the brain to help regulate abnormal signals that cause movement problems. While DBS is known to improve motor symptoms, its long-term effects on independence and overall quality of life are still debated.

In this study, we reviewed and analyzed data from 32 scientific studies including more than 2,500 people with Parkinson's disease who underwent DBS targeting the subthalamic nucleus (a specific brain region). We examined how DBS affected daily functioning and quality of life over time.

We found that DBS generally improves independence and quality of life within the first 6 to 12 months after surgery. Many patients experienced better mobility, greater ability to perform daily activities, and reduced need for medication. Some benefits were still present after two years. However, after five years, improvements were less clear and varied between individuals. This decline is likely related to the natural progression of the disease, especially cognitive and balance problems that are not fully improved by DBS.

Overall, DBS can provide meaningful short- and medium-term benefits, but long-term outcomes depend on disease progression and ongoing care.

## Introduction

Parkinson's disease (PD) is the second most common neurodegenerative disorder worldwide, following Alzheimer's disease.^1^ It currently affects over six million people globally,^2^ with projections estimating that more than 12 million individuals will be diagnosed by 2050.^3^ The prevalence of PD is approximately 107 per 100,000 individuals aged 50 to 59 years,^4^ and early-onset PD—before the age of 59—accounts for 32% of cases.^5^ Given this, PD is not uncommon among working-age individuals, significantly impacting their professional lives. In the United Kingdom, more than half (56%) of non-retired individuals with PD onset before the age of 50 were unemployed due to disability, with an average retirement age 4 to 7 years earlier than that of the general population.^6^ Beyond unemployment, PD also affects workplace productivity, leading to presenteeism (reduced efficiency while at work), increased absenteeism, and overall work impairment compared to healthy individuals.^7^ The economic burden of PD is substantial, with up to 49% of total costs in the United States and Europe attributed to indirect costs, such as reduced work capacity and early retirement.^8,9^ As a progressive and disabling disorder, PD affects both motor and non-motor functions.^10^ Motor symptoms include involuntary tremors, bradykinesia, postural instability, and severe difficulty in initiating movement. Meanwhile non-motor symptoms (NMS) encompass sleep-wake cycle disturbances, cognitive impairments such as memory loss, dementia, and hallucinations, as well as anxiety, alexithymia, mood disorders, orthostatic hypotension, autonomic dysfunctions, and sensory issues like pain and partial loss of smell.^11–14^ These symptoms contribute to a significant reduction in quality of life (QoL) that refers to a multidimensional concept encompassing at least three major domains: physical, mental, and social well-being. In the medical field, researchers and physicians often refer to health-related quality of life (HRQoL), which specifically examines how an illness or its treatment affects a patient's perceived health status, subjective well-being, and overall life satisfaction.^15^ Motor and non-motor symptoms affect also activities of Daily Living (ADLs) and Instrumental Activities of Daily Living (IADLs) which are two key categories used to evaluate a person's functional independence.^16^ ADLs refer to basic self-care tasks essential for everyday functioning, such as bathing, dressing, feeding, toileting, and walking. The ability to perform ADLs is fundamental for maintaining personal health and hygiene. Limitations in this area often indicate the need for assistance, home healthcare, or even placement in a residential care facility.^17^ In contrast, IADLs involve more complex activities that support independent living within the community. While not strictly necessary for survival, they are critical for autonomy and QoL. IADLs include tasks such as cooking, cleaning, laundry, managing finances, using transportation, and handling medications. These activities require higher levels of cognitive and physical functioning and are often assessed by occupational therapists in rehabilitation settings to determine the appropriate level of assistance.^18^ In summary, ADLs reflect basic self-care abilities, while IADLs indicate the capacity to manage life independently and safely in the community.

Given the impact of PD on daily functioning, managing both motor and non-motor symptoms is essential. To date, Levodopa (L-DOPA) remains the primary treatment for motor symptoms of PD, offering significant benefits, particularly in alleviating stiffness and akinesia.^19^ However, over time, its effectiveness diminishes, and patients often develop side effects, including motor fluctuations such as dyskinesia and wearing-off phenomena, along with non-motor fluctuations affecting autonomic function, cognition, psychiatric health, and sensory perception.^20–22^ Given these limitations, alternative nonpharmacological interventions have emerged, including deep brain stimulation (DBS), which has shown promise in alleviating symptoms and enhancing QoL.

High-frequency DBS of the subthalamic nucleus (STN) is a well-established surgical treatment for medication-refractory movement symptoms of PD. In 2005, the Movement Disorder Society (MDS) evidence-based medicine review update recognized STN-DBS as an effective symptomatic treatment for PD.^23^ STN-DBS is not a new treatment, as it has been used since the 1990s.^24^ Its benefits include improvements in motor symptoms and a reduction in the need for dopaminergic medication.^25,26^ DBS modulates abnormal activity in the basal ganglia, primarily targeting STN and the internal segment of the Globus Pallidus (GPi).^27,28^ Both targets are effective in improving motor symptoms, but STN-DBS is particularly known for significantly reducing the required levodopa dosage,^29^ thereby decreasing the medication burden and associated costs for patients.^30,31^

The STN plays a regulatory role by transmitting inhibitory signals within the cortico-subcortical networks of the basal ganglia.^32^ Ideal candidates for STN-DBS are patients with a confirmed diagnosis of PD for at least four years, without dementia, cognitive impairment, or severe depression, who experience motor complications, inadequate symptom control, and/or intolerance to medication.^33^

The main risks associated with the surgical procedure include intracranial hemorrhage and infection of the implanted hardware. Microelectrode recording (MER) is used to identify neuronal activation patterns in the STN and is currently the most commonly employed technique to assist in and validate target localization.^34^

Emerging evidence from long-term observational studies suggests that STN-DBS may delay certain late-stage disabilities, such as psychosis, falls, and the need for institutionalization, while also slightly extending survival compared to medically managed patients.^35^ However, motor improvement following DBS does not always correspond to an improvement in QoL; some patients have reported dissatisfaction despite experiencing enhanced motor function.^36^ Long-term studies with follow-ups exceeding five years have demonstrated the beneficial effects of STN-DBS on motor complications.^37^ However, findings on long-term QoL outcomes up to five years after DBS surgery remain inconsistent.^38,39^ There has been debate about the influence of sex on the effects of STN-DBS in PD. While short-term studies suggest similar motor and non-motor improvements in both male and female patients,^40–42^ findings on postoperative HRQoL remain inconsistent. The long-term impact of sex on STN-DBS outcomes is also unclear.

Given the ongoing debate regarding the impact of STN-DBS on QoL and ADLs, this systematic review/ meta-analysis aims to assess post-surgical changes in autonomy and QoL in patients with PD, relative to their pre-surgical baseline. By investigating the extent to which motor and non-motor improvements translate into sustained functional independence, this research seeks to clarify the role of DBS in enhancing overall patient well-being and long-term disease management.

## Materials and methods

**Figure 1.**
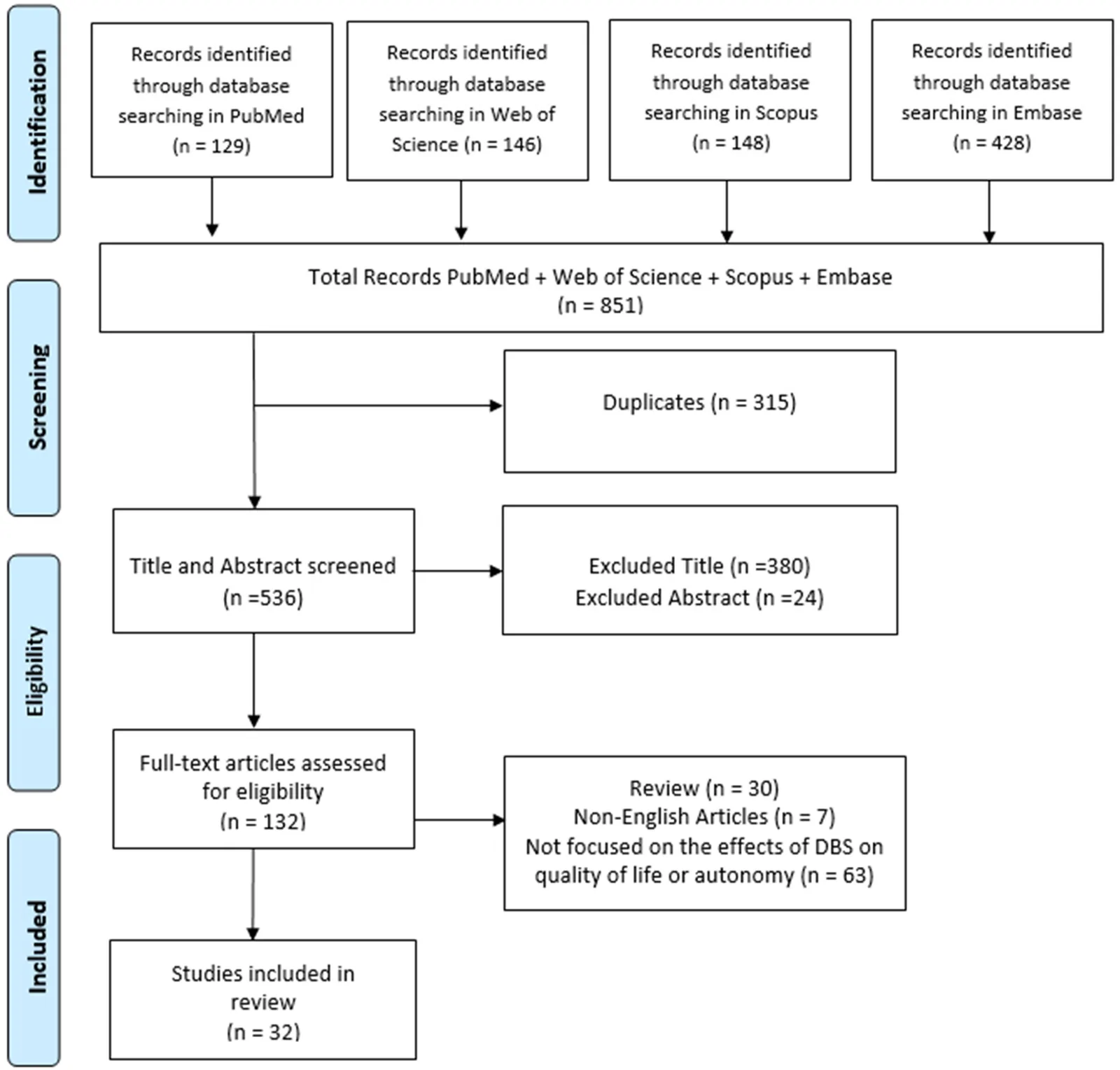
Key findings from included studies.

This systematic review/ meta-analyses was conducted and reported in accordance with the Preferred Reporting Items for Systematic Review and Meta-Analyses (PRISMA) (see Figure 1).^43^ A protocol for this review was registered on PROSPERO under the registration number CRD420251089887.

### PICO model

We employed the PICO (Population, Intervention, Comparison, and Outcome) model to shape our research question. Our target population comprises adults (>18 years) diagnosed with PD. The intervention under investigation is STN-DBS. In line with the design of the included studies and the analytical strategy of the meta-analysis, the comparison was defined as the preoperative or baseline condition of DBS-treated patients versus their postoperative follow-up assessments. The outcomes of interest included changes in QoL and activities of daily living following STN-DBS. By using this model, we aimed to systematically evaluate the impact of DBS on patients’ autonomy and overall well-being, providing a comprehensive understanding of its benefits in daily functioning.

### Search strategy

The studies were identified by searching in PubMed, Web of Science, Embase and Scopus databases in January 2025. All the studies fulfilling our selected criteria were evaluated for possible inclusion. The search combined the following terms: (deep brain stimulation) AND (parkinson)) AND (quality of life) AND (activities of daily living).

The search terms were identified for the title and abstract. After removing duplicates, all articles were evaluated based on title and abstract. This research was not restricted by the year of publication for the articles considered.

The inclusion criteria were: (i) studies including patients with PD who underwent DBS; (ii) Studies assessing QoL and activities of daily living; (iii) articles in English language only.

Exclusion criteria were: (i) reviews and meta-analyses; (ii) duplicated studies; (iii) single case study; (iv) conference proceedings, commentaries, letters to editor or book chapters (v) studies without mean or standard deviations in QoL or autonomy assessment.

### Study selection

To minimize bias and ensure a robust selection process, two authors (L.C. and M.C.D.C.) independently reviewed and extracted data from the studies. Any discrepancies were resolved through collaborative discussion and consultation with a third author (V.L.B). This multi-step approach guaranteed that at least three researchers independently assessed each article. In cases of persistent disagreement, all authors were involved in the final decision.

### Data extraction and analysis

The studies that met the inclusion criteria were summarized based on the following points: (1) Study characteristics: type of study and the country in which the data had been collected; (2) Patients characteristics: the sample size, age, gender, duration of disease, and education; (3) main and relevant findings.

Following the full-text selection, data were extracted from the included studies and reported in a table using Microsoft Excel (Version 2021). The extracted data included: study title, first author name, year of publication, study aims and design, sample size, type of participants, type of intervention and control, baseline performance, type of outcome and time-points for assessment, results, and key conclusions.

Moreover, the agreement between the two reviewers (L.C and M.C.D.C.) was assessed using the kappa statistic. The kappa score, with an accepted threshold for substantial agreement set at > 0.61, was interpreted to reflect substantial concordance between the reviewers. This criterion ensures a robust evaluation of the inter-rater reliability, emphasizing the achievement of a substantial level of agreement in the data extraction process.

### Risk of bias within individual studies

The risk of bias in the selected studies was independently assessed by L.C. and M.C.D.C. using the revised the Cochrane tool for non-randomised controlled studies-of exposures (ROBINS-E) tool, which comprises seven domains: (i) bias due to confounding, (ii) bias arising from measurement of the exposure, (iii) bias in selection of participants into the study (or into the analysis), (iv) bias due to post- exposure interventions, (v) bias due to missing data, (vi) bias arising from measurement of the outcome, (vii) bias in selection of reported result.

## Statistical analysis

The meta-analysis was performed using the R package ‘meta’ (version 4.5.1), setting the statistical significance level to α = 0.05. The main purpose of this analysis was to estimate the effect of deep brain stimulation (DBS) on patient recovery by comparing pre- and post-treatment changes. Next, we investigated whether these effects persisted over time. Thus, when multiple evaluations were reported, we considered all of them and grouped the studies according to their common follow-up periods (i.e., six months, one year, two years and five years).

As the outcomes were continuous and varied across studies, we extracted the statistical averages and standard deviations from the included studies, and the treatment effect of an intervention was estimated by pooling the standardized mean differences (SMDs) with 95% confidence intervals (CIs). In addition, we divided the studies according to the direction of the outcome. This involved grouping together studies in which a high score indicates a better condition and a low score indicates a worse condition (referred to as ‘increasing’), and vice versa (referred to as ‘decreasing’).

Heterogeneity was quantified by estimating the variance between I^2^ and τ^2^. The level of heterogeneity was higher than 75%, we considered the results obtained by the application of the random-effects model. Risk of bias was graphically investigated by funnel plots, and reported when appropriate.

## Results

### Synthesis of evidence

A total of 851 records were identified through database searches. After removing 315 duplicates, 536 records were screened by title and abstract. Of these, 380 records were excluded after title screening and 24 after abstract screening. A total of 132 full-text articles were assessed for eligibility, and 32 studies were included in the review and meta-analysis. The selection process is shown in Figure 1, whereas a description of the selected studies is provided in Table 1.

**Table 1.** Characteristics of the studies included.

Study	Study design	Simple size t0,t1	H&Y, Disease severity	Time follow up	Intervention	QoL questionnaire	Autonomy questionnaire	Cognitive, emotional and motor assessment	Results
Aviles-Olmos, 2014	Longitudinal observational cohort study	T0:41 T1 (5years): 41, (age 43.3 ± 9.8 41 females) T2 (8 years): 12 40.4 ± 9.8 (4 females)	Mean H&Y = 2.5 at baseline Disease duration T1: 12.9 ± 5.8 Disease duration T2: 12.3 ± 4	1, 5, and 8 years	STN-DBS	PDQ-39	UPDRS parts II (activities of daily living)	DRS-2, MMSE, BDI, BAI, UPDRS-III (motor examination), UPDRS-II (ADL), and UPDRS-IV (complications); AIDS	Long-term follow-up showed sustained gains in autonomy, whereas QoL improved at 1 year but returned to baseline by 5 and 8 years
Bezdicek 2022	Observational, pre–post intervention (prospective within-subject design)	T0: 32 (mean age 55.5 ± 7.8 years pre-surgery, 18 males) T1: 32 (56.95 ± 7.79)	Disease duration: 11.37 ± 3.67	1 year after STN-DBS (12.4 ± 0.9 months)	STN-DBS	IADL performance used as QoL proxy	PDAQ-15	BDI-II, MDR-UPDRS, DRS-2;	At 1 year post-STN-DBS, IADL improved, with cognition and LEDD as positive predictors and depression as a negative predictor.
Castrioto et al., 2022	Longitudinal observational study (monocentric), retrospective and prospective data collection	T0: 85 (52.4 ± 9 years, 29 females)	Disease duration: 12 ± 4.4	1 year and beyond 10 years after STN- DBS	STN-DBS	PDQ-37; PDQ-39 (only in two patients)	UPDRS II (ADL), Schwab & England ADL Scale (UPDRS VI)	BDI, MDRS	STN-DBS provided >10-year benefits in independence and QoL, with sustained ADL/PDQ-37 gains, progressive cognitive decline, and stable depression
Dafsari et al., 2016	Multicenter, open-label, prospective observational registry study	T0: 60 (35 male, mean age: 61.6 ± 7.8 years) T1: 60	H&Y 2.75	6 months	STN	ADL PDQ-8	MDS- UPDRSIII SCOPA ADL – Scale	NMSS	DBS at 6 months improved QoL, ADL, motor and non-motor symptoms; NMS gains correlated with QoL
Dafsari, Reker et al., 2018	Prospective, open-label, international multicenter observational study	T0 group Age ≤59 years n = 44 M/F 26/18 age: 53.2(65.3) T0 group Age 60 to 69 years n = 50 M/F 33/17 age: 64.7(62.4) T0 group Age ≥70 years n = 26 M/F 15/11 age: 72.3(62.4)	H&Y: 2.5 Disease duration 10.8 years 64.5	6 months	STN	PDQ-8	UPDRS-III SCOPA-ADL	MMSE	DBS improved QoL, ADL, motor function, and reduced LEDD; younger patients benefited most
Dafsari, Petry-Schmelzer et al., 2018	Prospective, open-label, multicenter observational study	T0 group: 50 M 31 aged 60.9 years (±8.3)	Hoehn&Yahr 2.5 Disease duration 10,3 years (±4,4)	6 months	STN	PDQ-8 ADL HADS	UPDRS-III SCOPA-B (Activities of Daily Living)	NMSS NMSQ SCOPA-A	6-month gains in QoL, ADL, motor function, and LEDD; non-motor benefits tied to anterior–medial–ventral stimulation.
Dafsari, Silverdale et al., 2018	Prospective, observational, international multicenter study	T0: M (50 males) age was 62.3 67 ± 8 years T1 group 5-Month follow-up: 67 T2 group 24-Month follow-up:67	Hoehn&Yahr 2.5 disease dura tion was 10.9 64 ± 8 years	5 and 24 months	STN	PDQ-8	NMSS attention/memory domain	SCOPA-A (motor exam), SCOPA-C (motor complications) NMSS NMSQ	At 24 months, STN-DBS improved QoL, ADL, motor and nonmotor symptoms, and reduced LEDD; QoL/ADL gains correlated with NMS reductions.
Dafsari, Weiß et al., 2018	Prospective, open-label, multicenter international observational study	T0 group: 88 patients 61.2 ± 8.1 years	Hoehn&Yahr: NA disease duration=10.5 years (SD = 4.5)	6 months	STN	PDQ-8	NMSS attention/memory domain	SCOPA-A (motor), SCOPA-C (motor complications), HADS	QoL, ADL, motor and LEDD improved; flat mood, pain, and urinary issues predicted greater gains.
Drapier et al., 2005	Observational, prospective cohort study	PD group: 27 M/F 19/8, years 60.8 ± 9.3	Disease duration of 14.6 ± 4.6 years	12 months	STN	PDQ-39 ADL MADRS	S&E	Mattis Dementia Rating Scale UPDRS Part I	Significant improvements in motor symptoms (UPDRS II and III, H&Y, S&E). Physical aspects of quality of life significantly improved. No significant improvement in emotional well-being, social support, cognition, or communication. L-Dopa equivalent dose reduced by 29%.
Dafsari et al., 2020	Prospective, observational, multicenter study	PD 73: M 47 61.9 years ± 7.7	H&Y: 2.5 Disease duration 10.4 years ± 5.0.	5 Months and 24 months	STN-DBS	PDQ-8	SCOPA-B (activities of daily living),	HADS-A HADS-D SCOPA-A (motor examination), SCOPA-C (motor complications)	DBS improved sleep, QoL, motor function, and reduced LEDD; sleep gains, partly sustained at 24 months, correlated with QoL.
Ferrara et al., 2010	Prospective observational cohort study	T0 group: 21 M/F 11/10 age 61.5 68 ± 6	Hoehn&Yahr 2.3	6 months and 12 months	STN - DBS	HRQoL PRQ-39 QLSM GDS SF-36 EQ 5D	UPDRS LFADLDS (Lange-Fahn ADL Dyskinesia Scale)	UPDRS III UPDRS Parts I–IV,	STN-DBS enhanced HRQoL in motor/health domains, not in life satisfaction; improvements correlated with ADL gains and less depression.
Fraix et al., 2005	Prospective multicenter observational study with double-blind motor evaluation at 3 months	PD group: 97 M/F 65/32, 43 ± 9 years	H&Y: NA Disease duration: 14 ± 5 years	6 months	STN	PDQL-37	UPDRS Part II, Schwab and England ADL Scale, LFADLDS	BDI Mattis Dementia Rating Scale	At 12 months, QoL, ADL, motor function, and LEDD (−59%) improved; cognition was stable apart from verbal fluency, with slight decline in motivation
Gorecka-Mazur et al., 2019	Prospective, observational monocentric study	PD group 25: F/M 5/20, 58.0 ± 9.0 years	H&Y: NA Disease duration: 10.9 ± 4.7	3, 6, and 12 months	STN-DBS	I-ADL WHOQOL-BREF SF-36	UPDRS Part II	MMSE UPDRS Part III (Motor Examination), UPDRS Part IV (Complications of Therapy)	At 3–6 months, HRQoL and ADL improved and were stable at 12; benefits included mobility, ADL, stigma, discomfort, and I-ADL, with strong correlation between I-ADL independence and HRQoL.
Jiang et al., 2022	Prospective observational study	T0: 27 T1: 23 PD group 2: M/F 16/7, 77.0 (75–85) years	H&Y: NA	1 year	STN	PDQ-39	Barthel Index (Activities of Daily Living, BI)	MMSE MoCA HAMD HAMA	DBS yielded 1-year improvements in motor, QoL, and ADL (46%, 38%, 67%) with smaller sustained benefits at ∼55 months. Sleep, mood, and LEDD (−57%) also improved. Minor complications occurred; no infections or deaths.
Jiang et al., 2023	Prospective observational cohort study	PD group: 53 F/M 29/24, Age of PD onset 49.53 (8.12)		1 year and 5 years post-surgery	STN-DBS	PDQ-39	UPDRS-II ADL	UPDRS- III SCOPA-A	DBS improved motor scores at 1 year (−40% UPDRS II, −37% III) with smaller benefits at 5 years; tremor remained stable. QoL gains faded by 5 years. LEDD fell 36% at 1 year, 15% at 5 years.
Jost et al., 2023	Prospective, observational, international multicentre study	Pd group Tremor- dominant: 33 F/M 10/23 (30.3/ 69.7), age 62.1 (9.0) PIGS group: 82 F/M 27/55 (32.9/67.1), age 63.0 (8.5)	H&Y: 2 Disease duration 10.5 years ±4.7	6 moths	STN-DBS	PDQ-39 PDQ-8 SI	ADL	UPDRS-III SCOPA-motor NMSS	People with PIGD improved in motor, non-motor, and QoL; tremor-dominant mainly in stigma/discomfort. Non-motor gains were greater in PIGD. LEDD reduced in both (−53% vs −47%)."
Jost et al., 2024	Prospective, observational, quasi-experimental, nonrandomized controlled trial	PD group: 108 (STN-DBS group: 62; MED group: 46) 63.7 ± 8.3 years	H&Y: NA Disease duration: 7.4 years	5 years	STN-DBS	PDQ-8 SI	SCOPA-ADL	UPDRS Part III SCOPA-M	Five years post-STN-DBS, QoL was stable with better mobility and fewer motor complications; MED patients declined. LEDD fell 62% with STN-DBS but rose 17% with MED. ADL gains correlated with QoL
Liu et al., 2019	Retrospective analysis of prospectively collected data (observational study)	PD group 45: M/F 21/24 age 61.80 ± 8.08	H&Y: 3.36 ± 0.86 Disease duration 143.00 ± 62.58	12 months	STN-DBS	PDQ-39	ADL	BDI-II UPDRS III MMSE	QoL improved in 51% of patients, with PDQ-39 gains across mobility, ADL, cognition, and discomfort. Benefits correlated with less depression/NMS; predictors were lower medication load, better baseline QoL, and earlier stage. LEDD −57%
Lyons et al., 2005	Prospective observational study	PD group: 59: M/F 44/15, age 59.5 69.8	Disease duration 11.9 65.0	12 months	DBS	PDQ-39	ADL UPDRS Part II	UPDRS Part III	UPDRS and PDQ-39 scores improved at 12 months and long term, with QoL gains linked to bradykinesia improvement. Medication use declined by 43% at 12 months and 53% long term
Nakamura et al., 2019	Prospective observational study	PD group: 21 age, 65.2 1.3 years;	H&Y: NA Disease duration, 12.2 0.6 years	12 months	STN	PDQ-39 HRQoL	UPDRS Part II ADL	MMSE FAB UPDRS Part III	DBS improved self-perceived continence (COPM), with no significant changes in gait or household work. LEDD and UPDRS-III off scores decreased, while PDQ-39 and cognition remained unchanged. Continence satisfaction correlated with pre-op HRQoL and post-op MMSE.
Nazzaro et al., 2011	Prospective observational study	PD group: 24 M/F 16/8, 64.2 6.5 years	H&Y: NA Disease duration was 10.6 3.5 years	12 months	STN	PDQ-39	UPDRS Part II ADL	UPDRS Part III	Non-motor symptoms decreased (12.1→7.3), with significant gains in PDQ-39, UPDRS II–III, and LEDD (−54%). QoL improvements were strongly linked to NMS reduction
Østergaard & Sunde, 2006	Prospective observational cohort study	PD group 26, 63 ± 8 years	H&Y = 3.7	1 year and 4 years post-surgery	STN	PDQ-39 HRQOL	UPDRS Part II ADL	MMSE UPDRS Part III and IV	Four years post-DBS: motor +55%, ADL +42%, dyskinesia −90%, off-periods −67%; axial symptoms worsened, off-motor stable. Dementia in 21%. LEDD −29%
Patel et al., 2003	Prospective observational study	PD group 16 56 ± 11 years	H&Y = 4.1 Disease duration was 10 (3.5) years	12 months	STN	PDQ-39	UPDRS Part II	UPDRS Part III	At 12 months: motor +61% (UPDRS-III), ADL +62% (UPDRS-II), LEDD −48%, dyskinesia/fluctuations ↓; PDQ-39 + 14%. Cognition stable/improved in 86%. Adverse effects minimal, mainly stimulation-related
Plaha et al., 2011	Prospective observational study	PD group 15: M/F 8/7, 65 ± 67.9 years	H&Y = NA Duration disease was 21.5613.5 years	31.7 months	Bilateral caudal Zona Incerta (cZI)	SF-36	Fahn–Tolosa–Marin Tremor Rating Scale – Part C	Fahn–Tolosa–Marin Tremor Rating Scale – Parts A and B	Tremor improved by 74% overall; SF-36 physical and mental by 24% and 22%. No tolerance up to 7 years. Few side effects (3 transient dysarthria, 1 infection).
Besse-Pinot et al., 2021	Prospective multicenter observational cohort (ancillary analysis of the PREDISTIM study)	Pre-opRBD + n = 242, M/F 169/73, 61.0 ± 7.2 years Pre-opRBD- n = 206, M/F 131/75, 59.5 ± 7.7 years	H&Y = NA Disease duration ≥5 years	12 months	STN	PDQ-39	MDS-UPDRS II PDQ-39 ADL subdomain	ASBPD MoCA SDMT	Patients with and without RBD showed comparable improvements in motor symptoms (UPDRS-III −52% vs −54%), ADL, LEDD reduction (−52% vs −49%), and QoL. No group differences were found in cognitive or behavioral outcomes at 12 months. Preoperative RBD did not predict poorer DBS outcomes
Pusswald et al., 2019	Prospective cohort study with Reliable Change Index (RCI) analysis	PD group: 118 M/F 57/61, 63.50(±7.66) years	H&Y = NA Disease duration 5 years	12 months	STN-DBS	SF-36	B-ADL	MMSE WST-IQ BDI-II FAI	DBS improved depression (42.9%), physical (44.4%) and mental health (50%). ADL improved in 10% and remained stable in 80%; subjective memory stable in 76.2%. Psychosocial outcomes were more stable or improved vs PD-BMT, MCI, and controls
Sharma et al., 2019	Prospective observational cohort study	PD group: 30, M/F 21/9, 77.5 ± 2.1 years	H&Y = NA Disease duration 11.8 ± 5.3	1 year and 2.5 years	STN-DBS	PDQ-39	UPDRS Part II (ADL)	MMSE MoCA UPDRS Part III (motor), Part IV (dyskinesia, OFF time)	DBS improved motor scores (−31% at 1 yr, −27% at 2.5 yrs), dyskinesia, OFF time, and LEDD (−48%, −54%). QoL/ADL unchanged; cognition stable with slight mentation decline. Post-op confusion in 36%; QoL stability likely influenced by comorbidities and axial symptom
Siderowf et al., 2006	Prospective longitudinal observational study	PD group: 18, 87% men, 57.33 ± 9.01 years	H&Y = NA disease severity measured with UPDRS-III (off meds): 41.9 ± 12.1 Disease duration 12 ± 3.8 years	6 and 35.9 months	STN-DBS	PDQ-39, SF-36, VAS	ADL	UPDRS-III and IV	Long-term follow-up showed sustained improvements in VAS (63%) and PDQ-39 domains (ADL −29%, well-being −26%, stigma −43%, discomfort −35%). Mobility and communication declined slightly from 6-month peak but remained above baseline. Dyskinesia (−56%), OFF severity (−33%), and medication (−25%) improved. PDQ-39 was more sensitive than SF-36. Strong short-term responders maintained long-term benefit
Straits-Tröster et al., 2000	Observational comparative cohort study (not randomized)	Pallidotomy: 23 M/F 11/12, 62.7(7.5) years Pallidal DBS: 9 M/F 6/3, 50.3(13.0) years Thalamic DBS: 7 M/F 6/1, 65.1(12.0) year	H&Y = NA Medically intractable Parkinson's disease with motor fluctuations, dyskinesias, and/or tremor-predominant type	3 months	Pallidal DBS: Globus Pallidus Internus (GPi) Thalamic DBS: Ventral Intermediate Nucleus (VIM) of the Thalamus	SIP PDQ-39	SIP Body Care & Movement, PDQ-39 ADL subscale	UPDRS III BDI BAI POMS	Pallidal DBS improved HRQoL (SIP), anxiety, and motor function (+31%), with trend-level depression improvement. Thalamic DBS yielded only modest motor benefit (+9%). Physical/psychosocial gains were greater with pallidotomy and pallidal DBS
Williams et al., 2010	Randomized, open-label controlled trial (RCT)	PD group: 183	H&Y = ≤2·0, 2·5, 3·0, and ≥4·0 Disease duration <5, 5–9, 10–14, and ≥15 years	12 months	STN	PDQ-39	PDQ-39 ADL domain, UPDRS Part II	UPDRS IV DRS-II PDQ-39 Emotional Well-being domain	Surgery outperformed best medical therapy for PDQ-39 (total, mobility, ADL, discomfort), UPDRS off, and dyskinesia; LEDD −34%. Mild verbal fluency/vocabulary decline. One surgery-related death; 19% serious AEs
Zahodne et al., 2009	Randomized controlled trial (RCT – COMPARE trial, NIH-funded)	PD group 42 (20 STN, 22 GPi): 61.3 years	H&Y = NA Disease duration: STN = 13.6 years GPi = 12.3 years	6 months	STN	PDQ-39	PDQ-39 ADL domain	MMSE, DRS-2, BDI-II UPDRS-III	QoL improved in GPi (−38%) > STN (−15%), with greater gains in mobility, ADL, stigma, social support. Both groups improved in well-being, cognition, discomfort; STN lacked communication gains. Depression predicted QoL improvement. STN had verbal fluency decline (−5.7), linked to communication QoL. No LEDD reduction
Cai et al., 2024	Retrospective observational cohort study	PD group. 94 M/F 62/32, 60.6 ± 6.8 years	H&Y = NA Disease duration: 152.4 ± 62.3	1 year, 5 years, and 10 years	STN-DBS	No formal QoL questionnaire used	MDS-UPDRS-II (motor ADL)	MDS-UPDRS-III	OFF time decreased at 1 year (−36.9%) and 5 years (−40.9%), but not at 10. Dyskinesia time was unchanged. LEDD fell by 41.4% at 1 year and 35.1% at 5. MDS-UPDRS II–III worsened at 5 and 10 years, with gradual ADL decline. Falls increased at 5 years, and dementia rose from 1.7% pre-op to 10.7%. Adverse events were mostly mild and device-related, infections being most common.

**Legend**: S&E = Schwab and England Scale; MADRS = Montgomery–Åsberg Depression Rating Scale; NMSS = Non-Motor Symptoms Scale; PDQ-8 SI = 8-item Parkinson's Disease Questionnaire Summary Index; SCOPA = Scales for Outcomes in Parkinson's Disease; UPDRS Part III = Unified Parkinson's Disease Rating Scale, Part III (Motor Examination); SCOPA-M = Scales for Outcomes in Parkinson's Disease – Motor, Activities of Daily Living (ADL), and Complications; PCS = Physical Component Summary; DRS-2 = Mattis Dementia Rating Scale, Second Edition; BDI = Beck Depression Inventory; BAI = Beck Anxiety Inventory; AIDS = Assessment of Intelligibility for Dysarthric Speech; PDAQ-15 = Penn Parkinson's Daily Activities Questionnaire; NMSQuest = Non-Motor Symptoms Questionnaire; UDysRS = Unified Dyskinesia Rating Scale; ASBPD = Ardouin Scale of Behavior in Parkinson's Disease; MMSE = Mini-Mental State Examination; FAB = Frontal Assessment Battery; FTM TRS = Fahn–Tolosa–Marin Tremor Rating Scale – Parts A (tremor), B (upper limb function), C (activities of daily living); MoCA = Montreal Cognitive Assessment; SDMT = Symbol Digit Modalities Test; B-ADL = Bayer Activities of Daily Living Scale; BDI-II = Beck Depression Inventory, Second Edition; FAI = Forgetfulness Assessment Inventory; MDS-UPDRS Part II = Movement Disorder Society – Unified Parkinson's Disease Rating Scale, Part II (Motor Experiences of Daily Living); FOGQ = Freezing of Gait Questionnaire; VAS = EuroQol Visual Analogue Scale; ADL = Activities of Daily Living; SIP = Sickness Impact Profile; SOFAS = Social and Occupational Functioning Assessment Scale; POMS = Profile of Mood States; PDQ-39 = Parkinson's Disease Questionnaire – 39 items; PDQL-28 = Parkinson's Disease Quality of Life Questionnaire – 28 items; PDSS-8/9 = Parkinson's Disease Sleep Scale, items 8 and 9.

STN- has been extensively studied for its impact on autonomy and QoL in PD patients. Across multiple studies, STN-DBS consistently improves motor function, enhances ADLs, and reduces medication dependence, leading to significant QoL benefits. However, the extent and sustainability of these improvements vary depending on disease progression, patient characteristics, and the presence of non-motor symptoms.

### Consistent findings across studies

The benefits of STN-DBS in enhancing autonomy and reducing disability are consistently supported by several studies, including those by Aviles-Olmos et al.(2014a),^44^ Bezdicek et al.,^45^ Castrioto et al.,^46^ Dafsari et al.^47–49^ Patients undergoing DBS experience substantial improvements in mobility, motor symptom relief, and stigma reduction, contributing to enhanced self-sufficiency. Notably, studies by Dafsari et al.^48,50,51^ and Bezdicek et al.^45^ highlight that DBS significantly enhances IADLs, such as household management and medication adherence, thereby reinforcing its role in sustaining long-term independence.

Beyond motor outcomes, DBS has also demonstrated positive effects on NMS, further strengthening its contribution to patient autonomy. Dafsari et al.^49^ reported significant reductions in fatigue, urinary dysfunction, and mood disturbances. In the same study, the long-term effects of STN-DBS on NMS were investigated, showing that improvements in sleep, fatigue, urinary symptoms, olfaction, and excessive sweating persisted for up to 24 months, supporting better QoL and daily autonomy. Nevertheless, some of these gains began to decline slightly after 5 months, underscoring the importance of continuous follow-up and individualized management of non-motor symptoms.

A broader perspective on psychosocial factors reinforces these findings. Straits-Tröster et al.^52^ demonstrated that both DBS and pallidotomy improve motor function, mobility, and ADLs, while also providing additional benefits in emotional well-being, anxiety reduction, and social engagement. However, they noted that while DBS enhances self-perception and physical independence, improvements in social relationships require additional psychological support. Similarly, Jost et al.^53^ reinforced the impact of DBS on non-motor symptoms, particularly in postural instability and gait difficulty (PIGD) patients, who experienced greater cognitive, urinary, and perceptual improvements compared to tremor-dominant patients. These findings suggest that DBS provides stronger non-motor benefits in certain PD subtypes, highlighting the need for individualized preoperative assessments.

In line with these results, Gronchi-Perrin et al.^54^ reported a 28% overall improvement in QoL six months after STN-DBS, with the most pronounced benefits in mobility (+32%) and stigma (+44%). However, communication worsened slightly, and patients retrospectively tended to overestimate their preoperative functioning, which obscured the real magnitude of improvement. These findings emphasize not only the effectiveness of STN-DBS in enhancing mobility and reducing stigma, but also the importance of prospective assessments to accurately capture patient-reported outcomes.

### Economic and functional benefits

The substantial improvements in autonomy following STN-DBS have also been associated with economic benefits. Fraix et al.^55^ reported that after 12 months, motor function improved by 57% (Unified Parkinson's Disease Rating Scale, UPDRS-III), daily activities by 48% (UPDRS-II), and overall QoL by 28% (Parkinson Disease Questionnaire, PDQL-37). Autonomy gains were reflected in the Schwab & England scale, which increased from 42.1% to 74.8%, while the number of caregiver-dependent patients dropped from 85 to 30. Importantly, medication use decreased significantly, with levodopa-equivalent doses reducing from 1240 mg to 506 mg, leading to fewer side effects. These changes translated into a substantial reduction in healthcare costs, which decreased from €10,087 to €1673 per patient over six months. As a result, STN-DBS emerged as a cost-effective intervention, with the procedure costs (€36,904) recoverable within an average of 2.2 years.

### Broader functional autonomy enhancements

Gorecka^56^ reinforced the role of DBS in enhancing ADLs and IADLs, particularly in areas such as food preparation, housekeeping, and laundry, whereas tasks such as medication and money management showed minimal improvement. The ability to perform routine tasks independently was further supported by findings from Nakamura,^57^ who observed that mobility and independence in daily activities, such as dressing, eating, and personal hygiene, improved significantly following DBS. These improvements were largely attributable to better motor function and reduced limb stiffness, which together decreased the need for caregiver assistance.

Complementary evidence comes from Nazzaro^58^ and Besse-Pinot et al.,^59^ who documented a significant reduction in non-motor symptoms one year after DBS surgery, with the average number of symptoms decreasing from 12 to 7. Notably, improvements were observed in autonomic functions, including reduced urinary urgency, constipation, and dizziness upon standing, all of which contribute to maintaining daily independence.

The impact of DBS on fine motor control and self-perceived QoL was further demonstrated by Plaha,^60^ who found that DBS significantly reduced total tremor scores by 73.8% and improved ADLs by 80%. Patients reported notable gains in fine motor tasks such as handwriting (81.9% improvement) and pouring liquids (71.7% improvement), leading to greater ease in routine activities. Self-perceived QoL also improved significantly, with the physical component of the Short Form Survey Instrument (SF-36) increasing by 23.7% and the mental component by 22.4%, reflecting enhancements in both physical well-being and emotional stability.

### Variability in long-term outcomes

While short-term QoL gains following DBS are well-documented, long-term studies indicate a progressive decline in autonomy, primarily due to disease progression and the emergence of levodopa-resistant symptoms. Williams et al.^61^ examined STN-DBS in combination with best medical therapy (BMT) versus BMT alone, showing that DBS led to a significant 5.0-point improvement in PDQ-39 scores after one year, compared to only 0.3 points in the BMT group. The most notable improvements were observed in mobility (−8.9 points, p = 0.0004), ADLs (−12.4 points, p < 0.0001), and bodily discomfort (−7.5 points, p = 0.004), reinforcing the superiority of DBS in maintaining functional independence. However, the study also found that DBS did not significantly impact emotional well-being, cognition, or social support, emphasizing the need for complementary interventions to optimize non-motor aspects of QoL. Long-term analyses by Aviles-Olmos et al.,^44^ Castrioto et al.,^46^ and Zhan^62^ confirmed that while STN-DBS provides initial improvements in QoL and ADLs, these benefits diminish over time, particularly after five to ten years.

Cognitive decline and the worsening of axial symptoms—including balance difficulties and falls—progressively reduce sustained autonomy, leading to greater caregiver dependence. Nonetheless, Castrioto et al.^46^ reported that 98% of DBS patients were still living at home more than a decade after surgery, suggesting that although disease progression compromises functional outcomes, DBS plays an important role in delaying institutionalization and preserving a degree of independence.

Identifying predictors of postoperative QoL is essential for optimizing patient selection. Liu et al.^63^ investigated QoL outcomes one year after bilateral STN-DBS and found that 51.1% of patients experienced improvements, 40% reported no change, and 8.9% experienced a decline. The most substantial benefits were observed in mobility, ADLs, cognition, and bodily discomfort. Better postoperative outcomes were associated with higher preoperative QoL burden, lower dopaminergic medication use, and earlier disease stages. The study also emphasized that reductions in depression and other non-motor symptoms were key drivers of QoL improvements, reinforcing the importance of comprehensive preoperative evaluations.

Moreover, the relationship between motor function improvements and QoL gains was explored by Lyons & Pahwa,^64^ who found a strong correlation between reductions in bradykinesia and improvements in QoL. However, their study also noted that improvements in tremor and rigidity had less impact on overall QoL, suggesting that bradykinesia plays a more significant role in determining functional autonomy post-DBS. Collectively, these findings highlight that while STN-DBS effectively enhances mobility and daily functioning in the short-to-medium term, long-term benefits are influenced by disease progression, cognitive function, and non-motor symptoms. Tailored post-surgical care, including rehabilitation programs, cognitive monitoring, and psychological support, is essential for maximizing sustained autonomy and well-being in PD patients undergoing DBS.

Several studies highlight that the benefits of STN-DBS on QoL and autonomy vary significantly depending on patient age and sex. Dafsari et al.^49^ and Jiang et al.^65,66^ consistently show that younger patients (≤59 years) derive greater QoL improvements compared to older individuals (≥70 years). While older patients still experience gains in activities of ADLs and stigma reduction, their cognitive decline and axial symptoms progress more rapidly, limiting long-term autonomy. Jiang et al.^66^ specifically report that while motor symptoms improved significantly at one year post-surgery (UPDRS-II: 39.71%, UPDRS-III: 36.83%), these effects diminished over time (18.83% and 22.75% improvement at five years, respectively). Similarly, the Hoehn & Yahr (H&Y) stage initially improved but eventually returned to baseline, although daily living activities remained better than preoperative levels. Medication use (LEDD) decreased by 35.32% at one year and by 15.26% at five years, confirming a sustained but diminishing effect.

These findings align with Sharma et al.,^65,67^ who specifically investigated DBS outcomes in patients over 75 years old. While a significant reduction in motor symptoms was observed at one year (UPDRS-III: −30.8%), long-term improvements in QoL and ADLs were less pronounced. Moreover, this age group exhibited higher rates of postoperative complications, including gait difficulties and transient confusion, highlighting the necessity for careful patient selection and postoperative management in elderly populations.

### Impact of psychological and social factors

In addition to age and sex, psychosocial factors significantly influence DBS outcomes. Pusswald et al.^68^ found that while 77.8% of DBS patients reported improved physical health and 61.9% experienced reduced depressive symptoms, subjective memory function remained unchanged. This suggests that while DBS enhances physical independence, it may not directly influence cognitive perception of autonomy, highlighting the importance of psychological interventions to optimize patient outcomes.

Postoperative apathy has been identified as a key determinant of QoL following DBS.

Psychosocial factors such as stigma reduction also contribute to QoL outcomes. Patel et al.^69^ found that improved mobility post-DBS reduced frustration and emotional distress linked to motor impairments, allowing patients to feel more confident in social interactions. This sense of self-sufficiency further enhanced QoL, particularly in terms of emotional well-being and social engagement. However, while DBS positively affects psychosocial functioning, limitations remain. Sharma et al.^67^ found that depression and anxiety continued to impact autonomy, reinforcing the necessity for comprehensive preoperative evaluations and long-term psychological support.

These findings are consistent with Siderowf et al.,^70^ who reported long-term gains in ADLs (+29%), mobility (+20%), emotional well-being (+26%), and stigma reduction (+43%). However, domains such as communication, cognition, and social support remained unchanged, indicating that while DBS effectively preserves physical autonomy, its impact on broader psychosocial functioning is more limited.

While the majority of studies support STN-DBS as an effective intervention for improving autonomy, concerns remain regarding the sustainability of its benefits, especially in relation to cognitive decline, speech deterioration, and the persistence of non-motor symptoms. Zahodne et al.,^71^ Zhan et al.,^62^ and Østergaard & Sunde^72^ collectively demonstrate that while STN-DBS results in substantial early improvements in motor function, ADL, and QoL for PD patients, the long-term sustainability of these improvements is challenged by disease progression. Cognitive decline, axial symptoms, and increased fall risks gradually limit the benefits of DBS as the disease advances.

For instance, Østergaard & Sunde^72^ reported a 42% improvement in ADLs, a 67% reduction in “OFF” periods, and a 90% decrease in dyskinesias over four years, contributing to a 29% reduction in levodopa equivalent daily dose (LEDD). However, 21% of patients developed dementia, and axial symptoms, such as speech impairments and gait disturbances, worsened, negatively impacting long-term autonomy. Similarly, Zhan et al.^62^ observed that while STN-DBS improved ADLs in the short term, these benefits declined after five years, with an increase in falls and higher prevalence of dementia. While motor complications and medication dependency continued to improve, the effects of DBS on cognitive-related autonomy in later disease stages remained limited, as indicated by the unchanged institutionalization rates.

In addition to these cognitive challenges, Zahodne et al.^71^ compared unilateral STN-DBS and GPi-DBS finding that GPi-DBS led to more significant overall QoL improvements. While both groups experienced motor and mood enhancements, GPi-DBS patients showed superior outcomes in mobility, ADLs, stigma reduction, and social support. In contrast, STN-DBS patients showed primarily improved stigma, but a decline in verbal fluency, which may have contributed to comparatively lower QoL gains, highlighting the different impacts of these two DBS targets.

Although DBS has shown effectiveness in reducing medication burden and motor fluctuations, studies such as Dafsari et al.^49^ and Ferrara et al.^73^ emphasize that DBS does not significantly improve social participation or occupational function. This suggests that although DBS addresses motor symptoms, additional rehabilitative and psychological interventions are essential to sustain QoL improvements over time. Moreover, Drapier et al.^74^ reported that mental and social aspects of QoL, including emotional well-being, cognition, social support, and communication, showed no significant improvement post-surgery, with some areas even worsening. This underscores a dissociation between physical and non-motor benefits of DBS, indicating that while DBS enhances physical autonomy, its effects on psychological and social well-being are more limited.

Sharma et al.^67^ further reinforced this observation, noting that persistent psychological symptoms such as depression and anxiety significantly restricted the full impact of DBS on autonomy. Both Drapier et al.^74^ and Sharma et al.^67^ underline the necessity of integrating comprehensive preoperative screening with long-term psychological support to maximize DBS benefits across all QoL domains.

Taken together, the evidence demonstrates that DBS provides consistent long-term benefits in physical autonomy, with Siderowf et al.^70^ reporting lasting improvements in ADLs (+29%), mobility (+20%), emotional well-being (+26%), and stigma reduction (+43%). However, areas such as communication, cognition, and social support remain less responsive to DBS, confirming that its impact on broader psychosocial functioning is limited.

These findings reinforce the idea that while DBS improves motor function and daily independence, a more holistic approach, including psychological care and social support, is needed to fully optimize the treatment's benefits.

### Meta-analysis results

Given that there was significant heterogeneity in almost all analyses, we considered the results of the random-effects models.

#### Meta-analysis on the effect of DBS on autonomy

**Figure 2.**
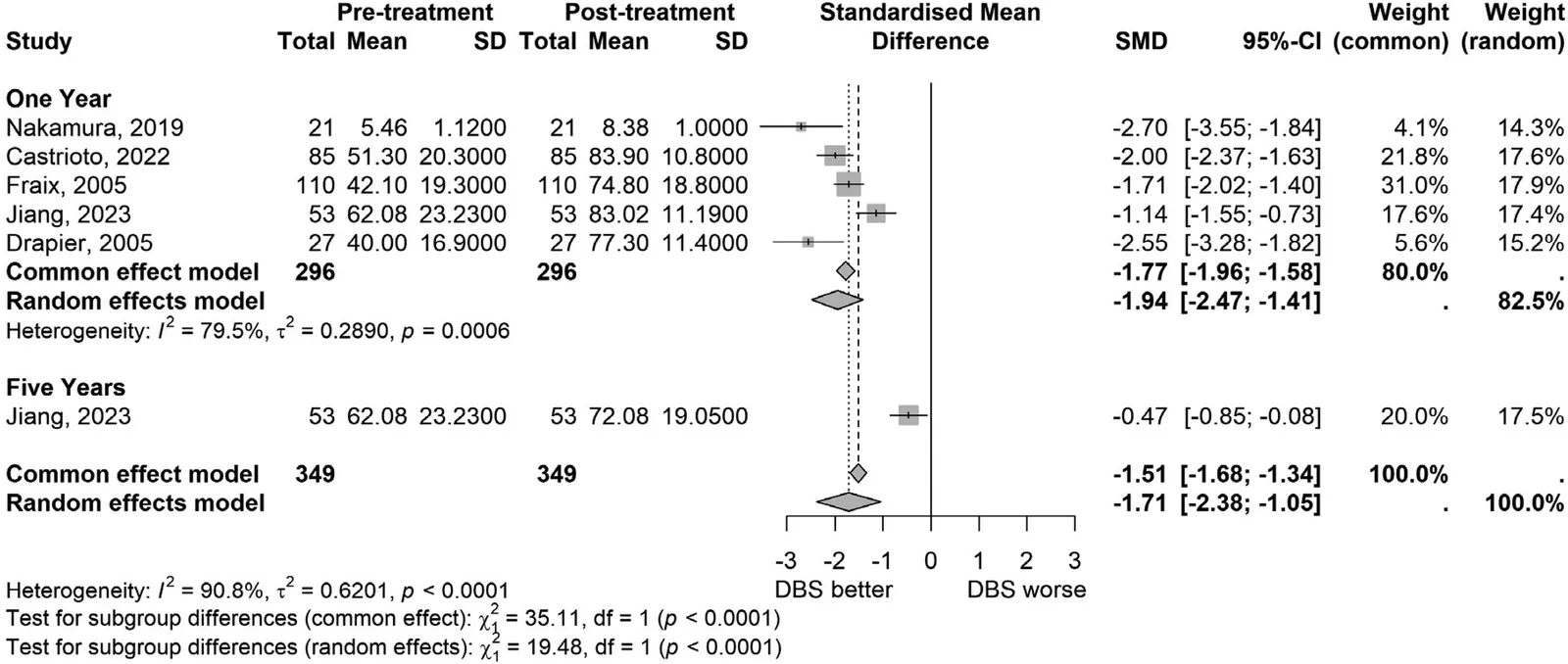
Comparison of the effects of DBS on autonomy, considering the studies in the increasing group (i.e., indicating an improvement as the mean value increases after the treatment), and subdivided according to the follow-up periods. Number of participants, with mean and standard deviation (SD) of changes in test score, is presented for each study in any group. The point estimate and the overall effect, with 95% confidence intervals, are indicated by the diamond in the bottom forest plot.

Figure 2 shows the results of meta-analysis including the studies in the increasing group (i.e., indicating an improvement as the mean value increases), and sub-grouped according to the time elapsed between the pre- and post-treatment periods. As can be seen, the overall estimate revealed significant heterogeneity across the studies (I^2^ = 90.8%, p < 0.0001). The high heterogeneity is probably due to the wide confidence intervals of^57,74^ studies in the one-year subgroup. However, despite the differences in effect size between studies, the trend always in favor of DBS treatment.

**Figure 3.**
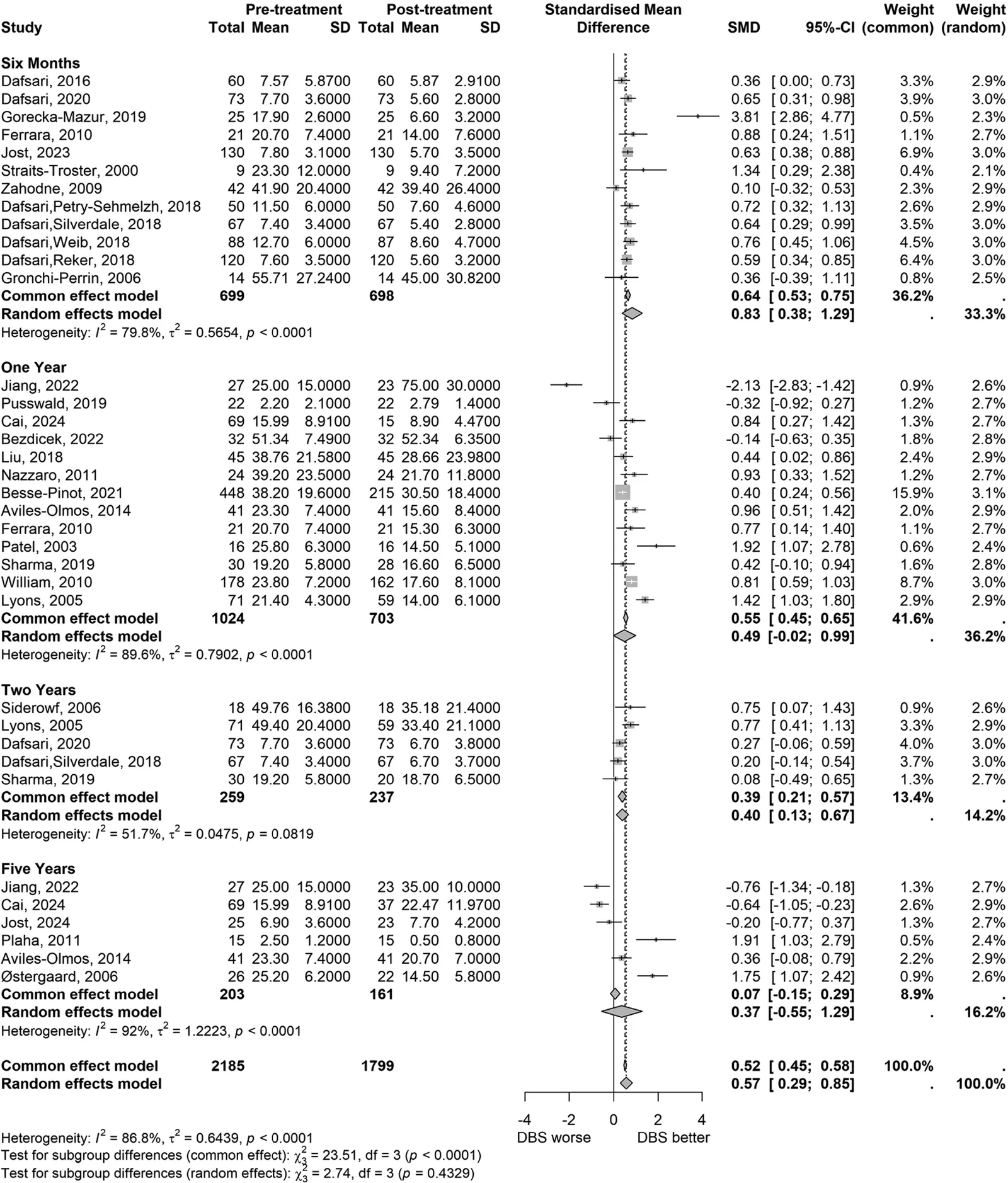
Comparison of the effects of DBS on autonomy, considering the studies in the decreasing group (i.e., indicating an improvement as the mean value decreases after the treatment), and subdivided according to the follow-up periods. Number of participants, with mean and standard deviation (SD) of changes in test score, is presented for each study in any group. The point estimate and the overall effect, with 95% confidence intervals, are indicated by the diamond in the bottom of forest plot.

**Figure 4.**
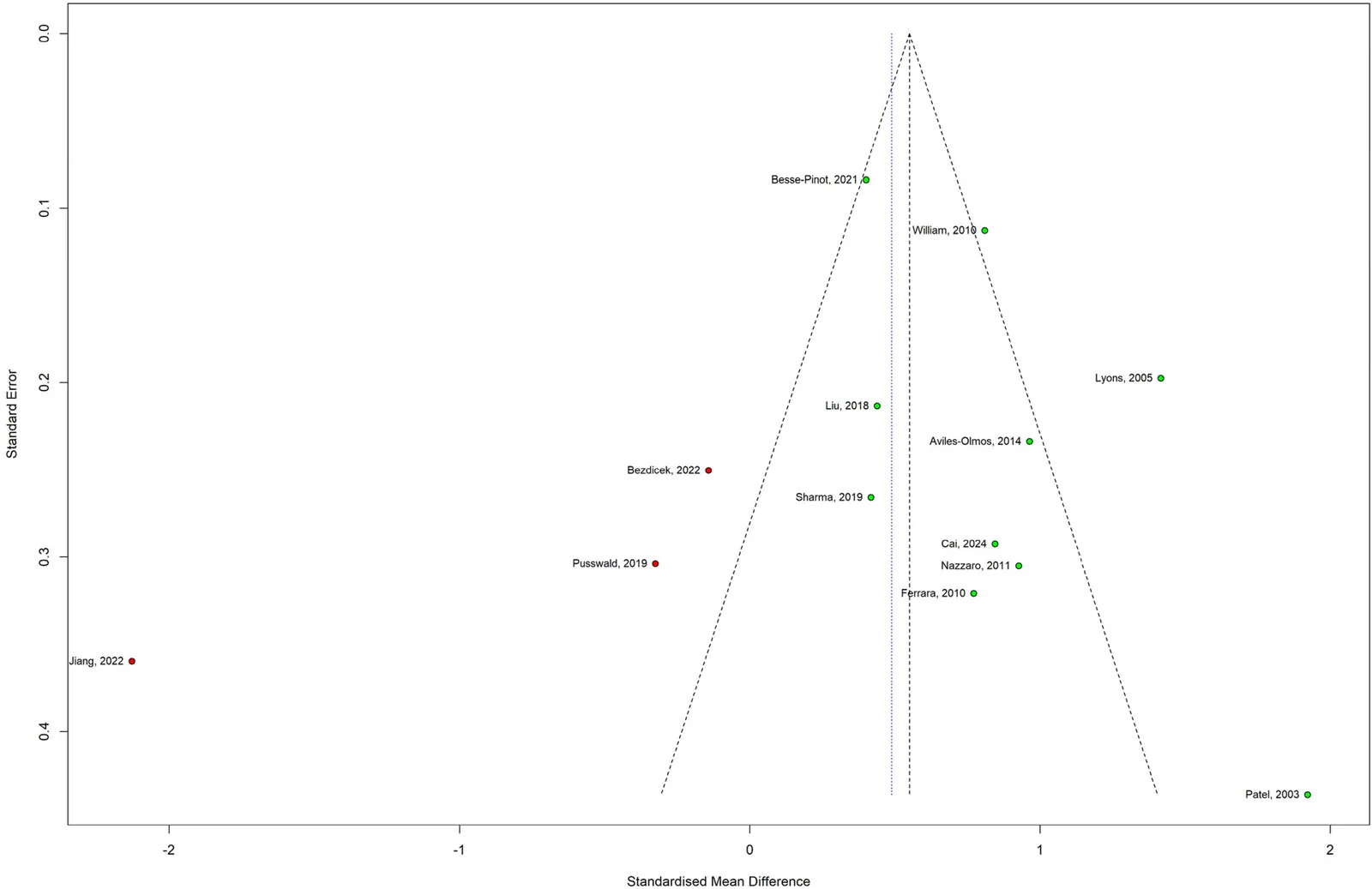
Funnel plot of the meta-analysis of the effects of DBS on autonomy, considering the studies in the decreasing group. It visually assesses the relationship between study effect sizes and their precision, often to detect potential publication bias. Studies opposing DBS are represented in red dots, while the studies in favor of DBS are represented in green.

**Figure 5.**
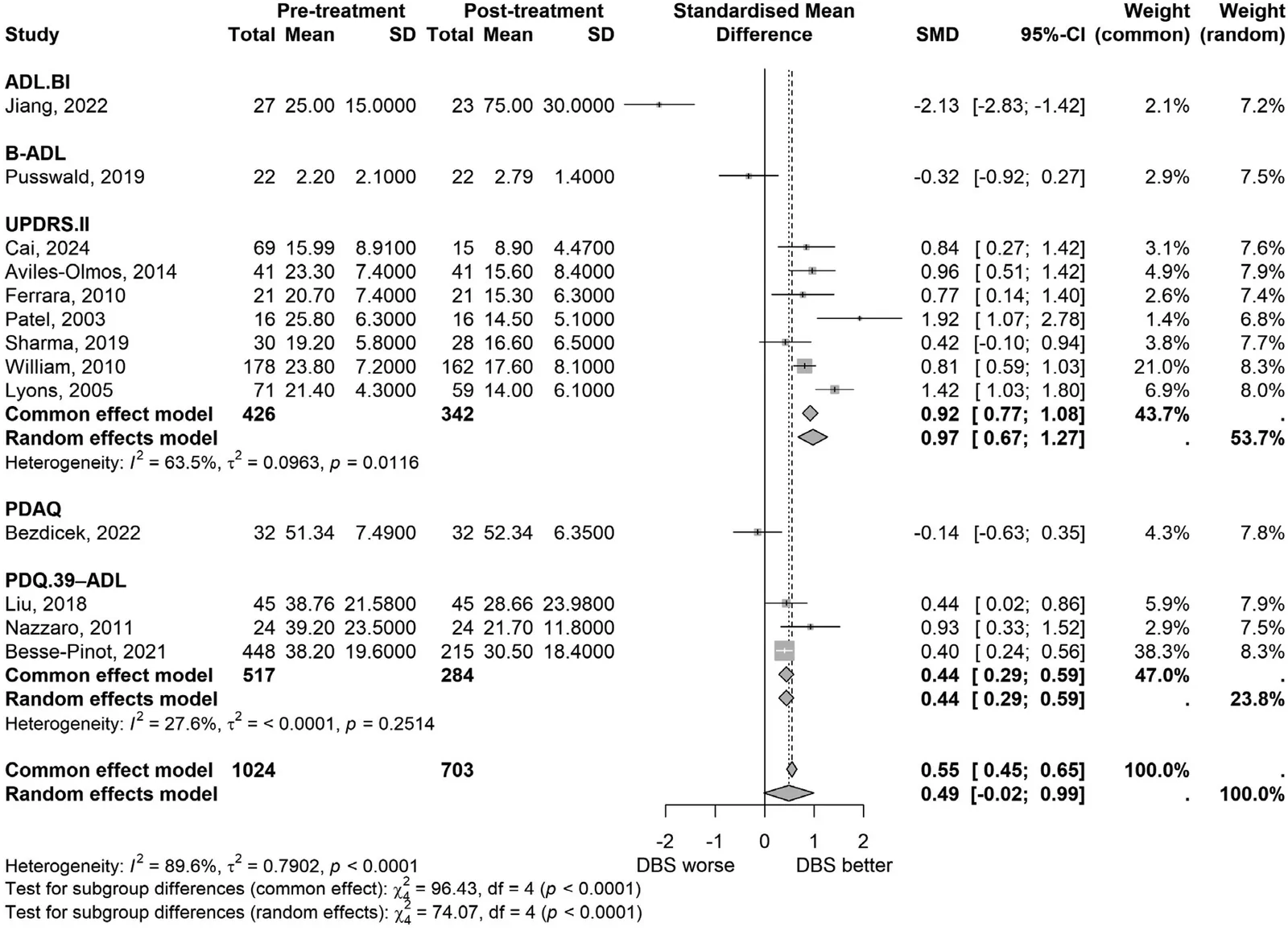
Comparison of the effects of DBS on autonomy at 1-year follow-up, considering the studies in the decreasing group and subdivided according to the outcome measure. Number of participants, with mean and standard deviation (SD) of changes in test score, is presented for each study in any group. The point estimate and the overall effect, with 95% confidence intervals, are indicated by the diamond in the bottom of forest plot.

We also noted a considerable amount of heterogeneity among the studies in the decreasing group (I^2^ = 86.8%, p < 0.0001), as shown in Figure 3. This was a general finding in all follow-up subgroups. The random effects model showed no significant differences among the follow-up subgroups (p = 0.4329), indicating homogeneity in terms of effects: despite the follow-up time, meta-analysis results were in favor of DBS treatment. In detail, at the 6-month follow-up, we found that all studies were in favor of the DBS treatment, although two studies did not reach the statistical significance.^54,71^ At the one-year follow-up, we found that the two studies with the largest sample sizes^59,61^ had the greatest weight in the common model (15.9% and 8.7%, respectively), as indicated by the larger squares in the forest plot, but not in the random model, which had high heterogeneity (I^2^ = 89.6%, p < 0.0001). This heterogeneity is also visible in the dispersion of points within the corresponding funnel plot in Figure 4. Its strongly asymmetrical distribution, mainly due to three studies opposing DBS,^45,65,68^ might lead the meta-analysis to overestimate the intervention effect. These studies showed a worsening in autonomy, deviating greatly from the trend seen in the other studies. However, when we performed the meta-analysis subgrouping the studies according to the outcome measure used, we found that these three studies employed different psychological measures; therefore, they cannot be directly compared with the other studies. On the contrary, we observed low heterogeneity in the subgroup of studies using the PDQ-39-ADL (I^2^ = 27.6%, p = 0.2514), all of which showed a significant effect size in favor of DBS (see Figure 5). Similarly, the subgroup of studies using the UPDRS II, despite the high heterogeneity (I^2^ = 63.5%, p = 0.0116) probably due to one insignificant study^67^ and the large 95%-CI of another study,^69^ had a global effect in favor of DBS treatment. The model built using data from studies with a two-year follow-up period, did not report high heterogeneity (I^2^ = 51.7%, p = 0.0819). Despite the lack of significance of one study,^67^ the meta-analysis estimated a small effect in favor of DBS treatment (see Figure 2). Finally, at the five-year follow-up, we observed the highest level of heterogeneity (I^2^ = 92%, p < 0.0001), with three out of six studies showing no statistical significance. The estimate of the intervention effect was not statistically significant, making it impossible to draw conclusions from this data.

#### Meta-analysis on the effect of DBS on quality of life

**Figure 6.**
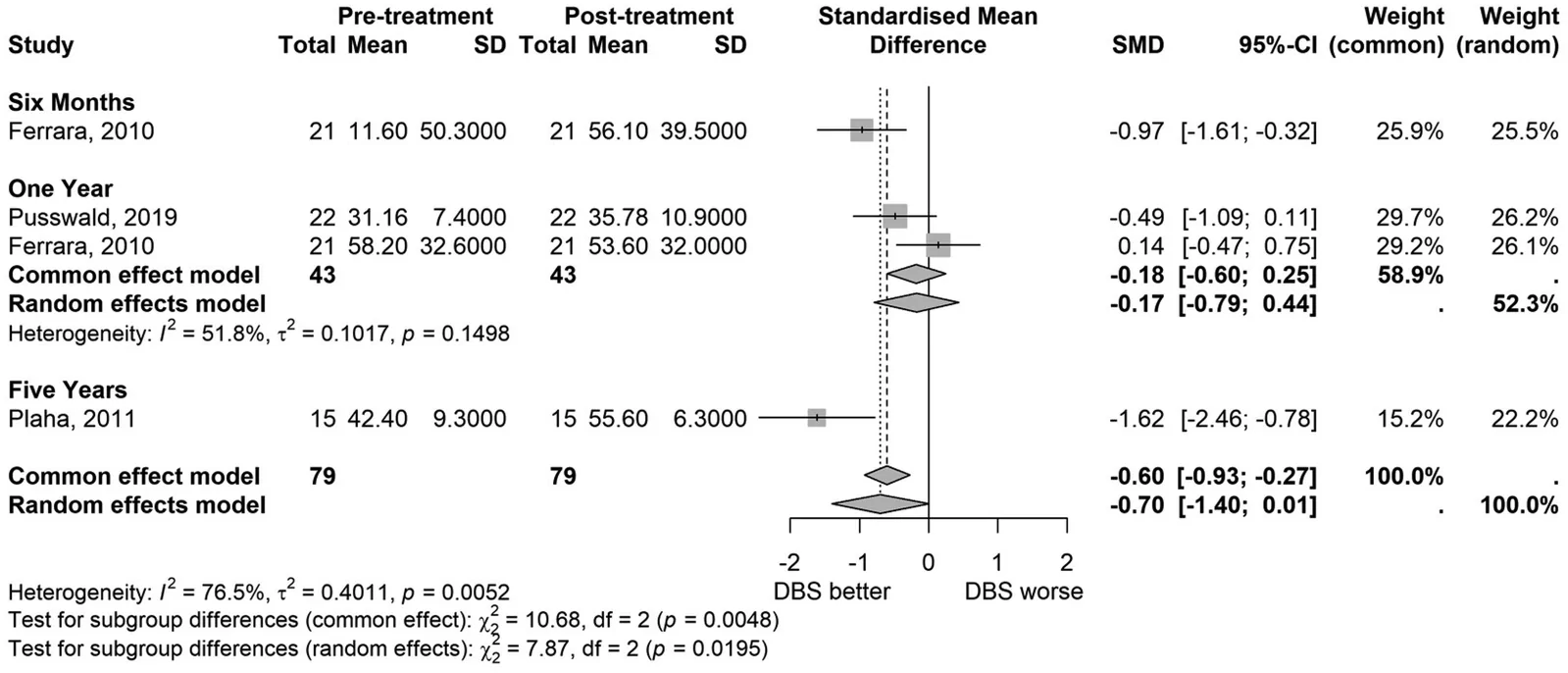
Comparison of the effects of DBS on QoL, considering the studies in the increasing group (i.e., indicating an improvement as the mean value increases after the treatment), and subdivided according to the follow-up periods. Number of participants, with mean and standard deviation (SD) of changes in test score, is presented for each study in any group. The point estimate and the overall effect, with 95% confidence intervals, are indicated by the diamond in the bottom forest plot.

Figure 6 shows the results of meta-analysis including the studies in the increasing group, and sub-grouped according to the time elapsed between the pre- and post-treatment periods. As can be seen, the overall estimate revealed a significant global heterogeneity (I^2^ = 76.5%, p = 0.0052), as well as among the follow-up subgroups (p = 0.0195). Approximately half of the weight in the random model is given by the subgroup with one-year follow-up (52.3%), including a study in favor of SBS treatment^68^ and a study opposing it.^73^ However, despite the differences in effect size between studies, the overall estimate was in favor of DBS treatment.

**Figure 7.**
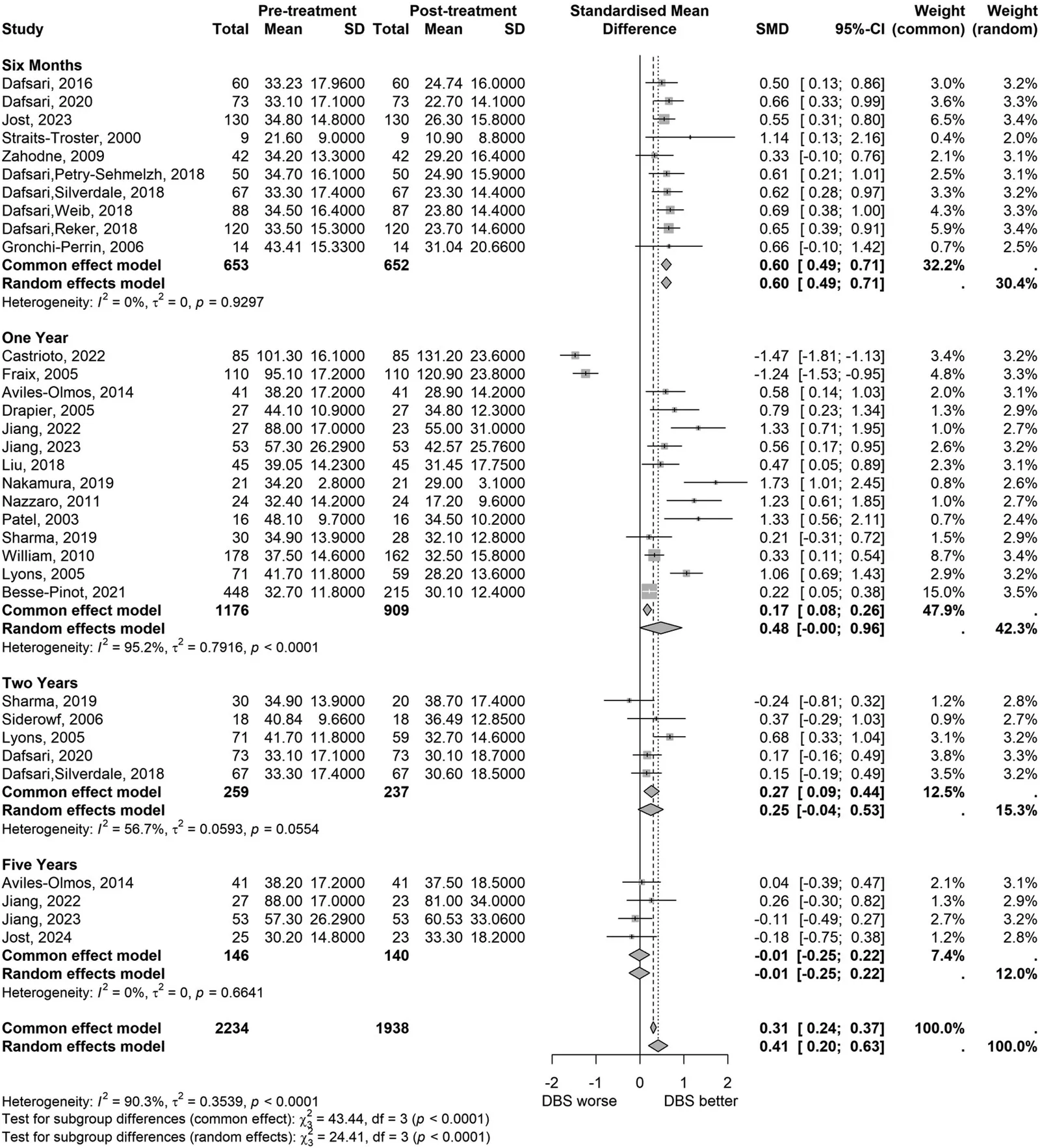
Comparison of the effects of DBS on QoL, considering the studies in the decreasing group (i.e., indicating an improvement as the mean value decreases after the treatment), and subdivided according to the follow-up periods. Number of participants, with mean and standard deviation (SD) of changes in test score, is presented for each study in any group. The point estimate and the overall effect, with 95% confidence intervals, are indicated by the diamond in the bottom of forest plot.

We also observed a considerable amount of heterogeneity among the studies in the decreasing group (I^2^ = 90.3%, p < 0.0001), as shown in Figure 7. Moreover, the random effects model showed significant differences among the follow-up subgroups (p < 0.0001). Indeed, at the 6-month follow-up there was an absence of heterogeneity (I^2^ = 0%, p = 0.9297), with an overall effect size estimation strongly in favor of DBS treatment. At the one-year follow-up, we found two large studies with results opposing DBS treatment,^46,55^ and one insignificant study.^67^ Thus, the heterogeneity was very high (I^2^ = 95.2%, p < 0.0001), and the overall estimate of the effect in this subgroup was slightly favorable to DBS treatment. As previously observed for autonomy, the two studies opposing DBS used a different outcome measure than all other studies and their results affected the asymmetry of the funnel plot, suggesting strong publication bias. At two-year follow-up period, we found a moderate heterogeneity (I^2^ = 56.7%, p = 0.0554). Due to the lack of significance in several studies, the meta-analysis indicated a slight effect in favor of DBS treatment. At the five-year follow-up, we observed an absence of heterogeneity (I^2^ = 0%, p = 0.6641). However, the global effect size was unclear this time, as none of the four studies in this subgroup were statistically significant.

## Risk of bias

**Figure 8.**
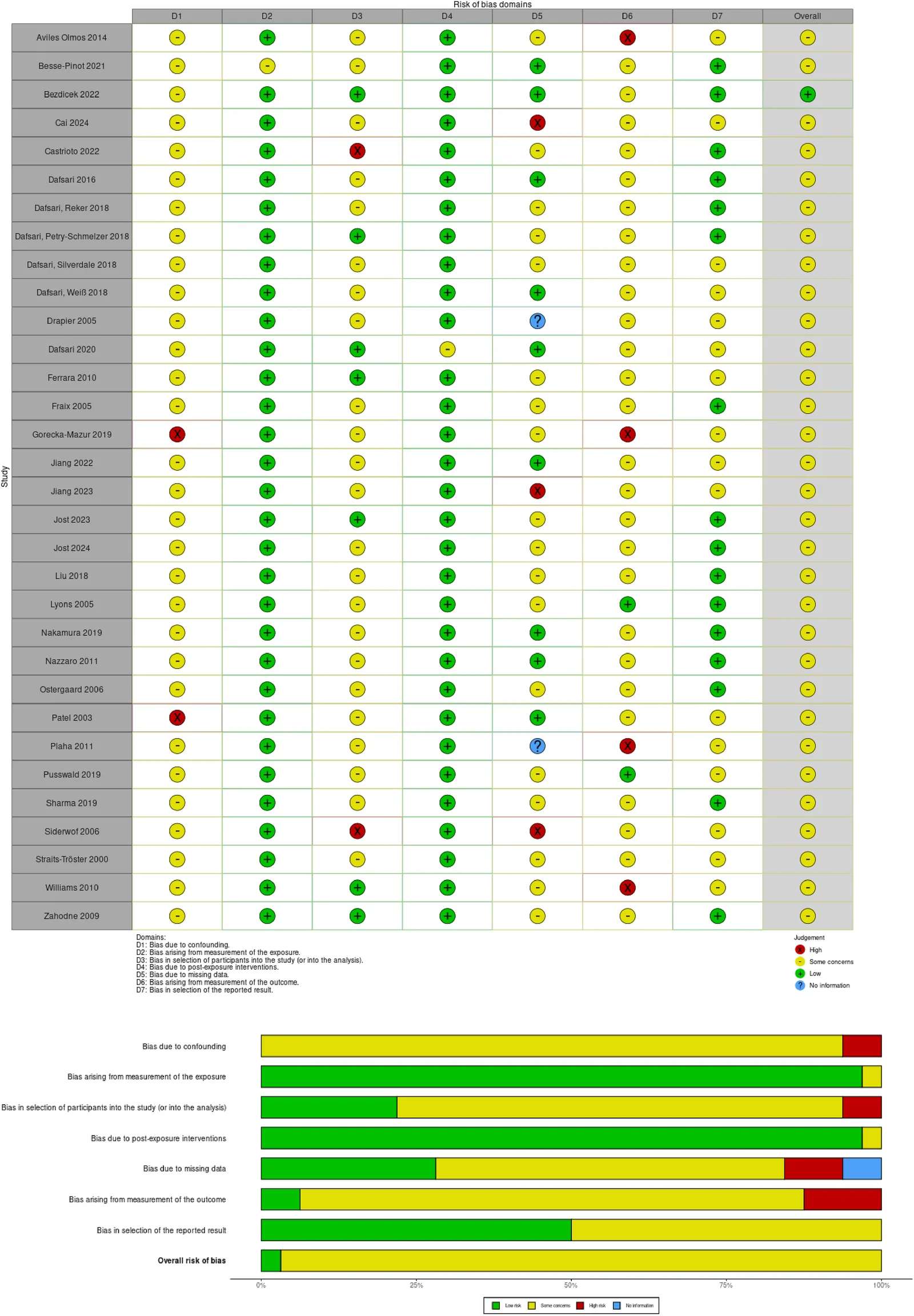
Shows the risk of bias (ROBINS-E) of studies regarding the impact of DBS on QoL and autonomy.

The Risk Of Bias In Non-randomized Studies - of Exposures (ROBINS-E) tool was used to evaluate the methodological quality of the included studies. Figure 8 summarizes the risk of bias across the seven domains, providing a visual representation of the distribution of judgments.

Across all studies, there were concerns regarding bias due to confounding, with three studies^56,69,75^ rated as having a high risk of bias in this domain.

For bias arising from measurement of the exposure, almost all studies were judged to have a low risk of bias, with the exception of one study.^59^

Regarding bias in the selection of participants into the studies, two studies^46,76^were judged to be at high risk of bias**.** Seven studies^45,49,61,71,73,77,78^ were instead judged to have a low risk of bias in this domain.

For bias due to post-exposure interventions, almost all studies were considered at low risk of bias, with the exception of Dafsari (2020), which was judged to raise some concerns.

Concerning bias due to missing data, three studies (Cai et al., 2024a; J.-L. Jiang et al., 2023b; Siderowf et al., 2006a) were rated as high risk, while 19 studies (Aviles-Olmos et al., 2014; Castrioto et al., 2022; Dafsari et al., 2018a, 2019a; Diaz et al., 2016; Ferrara et al., 2010a; Fraix, 2006a; Gorecka-Mazur et al., 2019a; S. T. Jost et al., 2023, 2024; Liu et al., 2018b; Lyons & Pahwa, 2005; Østergaard & Aa. Sunde, 2006; Pusswald et al., 2019a; Sharma et al., 2019a; Straits-Tröster et al., 2000; Williams et al., 2010b; Zahodne et al., 2009) showed some concerns, and the remainder were judged at low risk.

For bias in measurement of outcomes, four studies^44,56,60,61^were judged to have a high risk of bias, while three^68,75,81^were considered low risk, with the rest showing some concerns.

Finally, regarding bias in the selection of reported results**,** the majority of studies were rated as having some concerns**.** However, 16 studies^45–47,49,55,57–59,63,67,71,72,78–81^ were judged to have a low risk of bias in this domain.

## Discussion

This meta-analysis aims to examine the contribution of STN-DBS to patient QoL and autonomy, focusing on its broader implications for overall well-being and long-term management of PD.

Across the included studies, STN-DBS has consistently demonstrated significant improvements in motor function, QoL, and autonomy in patients with PD. However, while the short- to mid-term benefits of DBS are well established, long-term outcomes remain variable, influenced by factors such as disease progression, cognitive function, and psychosocial variables. Most studies included in the meta-analysis conducted follow-up assessments after 1 year and 6 months. A few studies reported follow-up periods of 2, 4, 5, or 7 years, and only two studies included follow-up after 10 years.

The studies reviewed confirm that bilateral STN-DBS remains a safe and effective treatment for patients with advanced PD, particularly those experiencing severe levodopa-induced dyskinesia and motor fluctuations in the medication-on state. The improvements in motor function are consistent with previous studies.^82–85^ Importantly, individuals with advanced PD experience not only disabling motor symptoms, but also a profound reduction in QoL, driven by limitations in ADLs, social functioning, and autonomy, often exacerbated by pharmacological side effects.^86^ While the therapeutic value of bilateral STN-DBS has been acknowledged for nearly 25 years,^83–85^ few studies have focused specifically on QoL and autonomy outcomes, compared to the extensive literature evaluating motor symptoms using the UPDRS. Nevertheless, QoL is increasingly recognized as a crucial endpoint, offering a more comprehensive evaluation of treatment effects in progressive neurodegenerative disorders such as PD. Several authors have recommended that QoL measures should become central outcomes in future surgical trials.^87,88^

## DBS and functional autonomy: sustained benefits with some limitations

Multiple studies have shown that STN-DBS enhances ADLs primarily by improving motor control and reducing dependence on dopaminergic medication. Significant gains in motor function, mobility, and autonomy have been reported, along with substantial reductions in levodopa equivalent daily dose (LEDD) and medication-related side effects.^55,89^ According to the literature^56,57,90^ these improvements translate into decreased disability and greater self-sufficiency in tasks such as personal hygiene, dressing, and household chores. Notably, improvements in motor symptoms following STN-DBS have also been associated to enhanced HRQoL, particularly within the social functioning domain.^91^

Despite these early benefits, long-term studies indicate a progressive decline in autonomy, especially five to ten years post-surgery as reported in the literature.^44,46,62,92^

While short-term HRQoL improvements are well documented,^76,86,93,94^ several studies have found that PDQ-39 scores tend to regress toward baseline levels by years 5 to 8. Sustained benefits tend to be observed in the PDQ-39 subdomains related to mobility, ADLs, stigma, social support, and bodily discomfort, whereas more pronounced declines are typically seen in emotional well-being, cognition, and communication.^44^ This long-term decline in HRQoL likely reflects the emergence of levodopa-resistant symptoms such as postural instability, cognitive impairment, and speech difficulties. The review by^92^ provides further evidence that motor improvements are generally maintained for up to 10 years, although the effectiveness of DBS progressively declines for bradykinesia and axial symptoms. Dyskinesias and motor fluctuations remain well controlled, but gait and speech impairments often emerge as new challenges, underscoring the need for complementary rehabilitation strategies to maximize long-term outcomes.

Similarly, short-term improvements in HRQoL observed within the first 3–6 months^86,95,96^ have often been attributed to reductions in depressive symptoms. LaGrange et al.^93^ found significant enhancements across all domains of a PD-specific QoL scale, particularly in relation to motor improvement in the medication-off state. Similarly, Lezcano et al.^38^ found that, while short-term benefits were consistent with other findings, longer-term effects (up to 2 years) were more pronounced and sustained in certain patient populations.

As PD progresses, the therapeutic effect of DBS is increasingly challenged by symptoms not responsive to dopaminergic therapy, including gait instability, falls, and cognitive decline. Although motor fluctuations and dyskinesias remain well-controlled, these emerging symptoms increasingly affect autonomy and limit the long-term benefits of DBS. This underscores the importance of long-term follow-up, the integration of multidisciplinary care, and the implementation of rehabilitation programs tailored to evolving patient needs.

Across the selected studies, different instruments were employed to assess QoL, including the PDQ-39, PDQ-8, and SF-36, contributing to methodological variability and heterogeneity in reported outcomes.

The study by Sideworf et al.^70^ further highlights that the PDQ-39, a PD-specific instrument, is more sensitive than the generic Short Form Health Survey 36 (SF-36) in detecting DBS-related changes in HRQoL. While small improvements were seen in SF-36 scores at 6 months, these were not maintained over time, underscoring the greater specificity and relevance of PDQ-39 in capturing disease-related impairments.^97^ This divergence between instruments provides a more nuanced picture: while DBS leads to clinically meaningful improvements in HRQoL when measured with PD-specific tools, broader aspects of disability may persist, as reflected by modest changes in generic scales. The choice of assessment tool therefore plays a critical role in determining the extent and nature of perceived benefit following DBS.

Overall, while STN-DBS yields significant early improvements in both motor function and QoL, its long-term efficacy is increasingly limited by the emergence of non-dopaminergic symptoms. These findings underscore the importance of personalized rehabilitation, long-term follow-up, and the use of disease-specific assessment tools to accurately capture patient-reported outcomes.

## Short- and long-term effects of STN-DBS on quality of life and functional autonomy in Parkinson's disease

Although DBS is primarily recognized for its effects on motor symptoms, its impact on NMS is increasingly acknowledged. Studies by Dafsari et al.^49^ and Witte et al. (2017) suggest that DBS can alleviate symptoms such as fatigue, urinary dysfunction, and mood disturbances, contributing to enhanced QoL. However, the extent of these improvements varies. For example, literature suggests that^98^ urinary dysfunction remained a persistent issue even three years post-surgery, suggesting that while DBS can address certain NMS, it does not offer a comprehensive solution for all aspects of PD. Evidence supporting the beneficial effects of STN-DBS on NMS has emerged from both clinical rating scales and objective laboratory assessments. Improvements have been reported in depression,^99^ anxiety,^94^ and pain,^100^ as well as in sleep (via polysomnography),^101^ gastrointestinal function (13CO_2_ excretion),^102^ urinary symptoms (urodynamic studies),^103^ and orthostatic regulation (tilt-table testing).^104^

Psychological factors also play a crucial role in determining post-DBS QoL. According to the literature^105^ postoperative apathy has been shown to significantly diminish patient's perceived benefits, despite comparable motor improvements between apathetic and non-apathetic patients.^106^ This underscores the importance of preoperative psychological screening to identify individuals who may require additional support to maximize QoL gains.

Data on the prevalence of apathy in the early postoperative period after STN-DBS are limited, ranging from 23% to 60%.^107–109^ Several studies have reported an overall increase in mean apathy scores following the procedure.^110,111^ The variability in prevalence can be explained by the existence of different forms of apathy with distinct causes and treatments. Early postoperative apathy is often the results from dopaminergic withdrawal due to reduced medication intake after surgery, which reveals presynaptic degeneration of mesolimbic dopaminergic terminals.^107^ This form of apathy, typically occurs within the first year, is influenced by drug management and stimulation parameters and may last up to 12 months. In contrast, long-term dopa-resistant apathy can develop as part of a dysexecutive syndrome linked to the progression of α-synucleinopathy.^112,113^

Another common postoperative finding is a decline in verbal fluency, which can compromise communication skills. In patients experiencing apathy, this decline be further exacerbated by reduced self-activation, limiting the motivation to initiate conversation.^94,114^

Thus while DBS can enhance physical independence, improvements in the subjective perception of autonomy are not always observed, underscoring the need for integrated psychological and rehabilitative.^67,68^

The impact of DBS on social participation and occupational functioning appears variable. Some studies suggest improvements in psychosocial well-being,^106^ whereas others indicate that DBS does not consistently result in enhanced professional involvement or social engagement.^73^ These observations indicate that, although physical autonomy tends to improve, additional interventions may be required to support long-term psychosocial outcomes.

### Variability in outcomes: influence of age, sex, and disease stage

Patient characteristics, including age and sex, significantly influence DBS outcomes. Studies consistently indicate that younger patients (≤59 years) experience greater long-term benefits in QoL and ADLs compared to older patients (≥70 years).^49,65–67^ While older patients still demonstrate functional improvements, they also experience more rapid cognitive decline and axial symptom progression, limiting their long-term autonomy. In particular, Jiang et al.^66^ observed that motor symptoms improved significantly one year post-DBS, but these benefits gradually declined over time, emphasizing the need for age-specific postoperative care.

Sex-related differences in DBS outcomes have also been investigated, although findings remain inconclusive. Some studies suggest that men may experience more favorable long-term outcomes. For instance, Kim et al.^115^ reported greater long-term improvements in physical QoL among male patients, indicating that sex-specific factors may influence therapeutic efficacy. Other evidence, however, points in the opposite direction. Hariz et al.^116^ reported that STN-DBS led to significant HRQoL improvements exclusively among female PD patients, contrasting with other studies suggesting comparable benefits across sexes.^42^ These discrepancies may be partly explained by methodological variability, particularly the use of different HRQoL assessment tools, with PD-specific instruments potentially capturing sex-related differences more sensitively than generic ones. Additionally, previous research has shown that men generally report better physical HRQoL than women, a trend also observed in stroke populations.^117^

The reasons behind sex differences in postoperative physical HRQoL remain unclear, especially given that motor improvements after DBS are generally similar across sexes. Several explanations have been proposed. Women with PD appear to have a higher prevalence of musculoskeletal problems, such as joint and bone disorders, which persist even after DBS and contribute to functional impairment.^118^ These issues have been identified as a major cause of reduced HRQoL, particularly in women. Moreover, women may be more susceptible to emotional distress related to disease progression, which can negatively impact physical HRQoL. Taken together, these findings suggest that sex-related differences in postoperative outcomes likely reflect a complex interplay of biological, psychosocial, and methodological factors.

### Cognitive decline and long-term challenges

Although DBS has demonstrated robust efficacy in improving motor symptoms in PD, its long-term impact on cognitive functioning remains less clear. A growing body of evidence points to concerns regarding postoperative cognitive decline, particularly affecting verbal fluency and executive functioning.^62,71,72^ For instance, Østergaard & Sunde^72^ reported that 21% of patients developed dementia over time, while Zhan et al.,^62^ found increased rates of cognitive impairment and falls five years after STN-DBS, contributing to reduced autonomy in daily functioning.

These findings underscore the importance of long-term cognitive monitoring in patients undergoing DBS, whilealso highlight the need to differentiate between changes driven by the natural progression of PD and those potentially influenced by DBS itself.

Comparative studies further suggest that target selection may play an important role in shaping cognitive outcomes. GPi-DBS has been associated with more favorable cognitive and psychosocial profiles compared to STN-DBS. For example, patients receiving GPi-DBS have shown greater improvements in mobility, ADLs, stigma reduction, and social support, while those treated with STN-DBS were more likely to experience a decline in verbal fluency.^71^ These findings support the adoption of personalized targeting strategies to balance motor, cognitive, and QoL goals when determining the optimal stimulation site.

However, it is crucial to interpret long-term cognitive and functional outcomes with caution due to possible preoperative selection bias. DBS candidates are typically chosen based on favorable characteristics—such as younger age at onset, strong levodopa responsiveness, and preserved cognition—which may inadvertently exclude individuals at higher risk for cognitive decline. This may lead to an overestimation of long-term cognitive stability associated with DBS. Additionally, cultural and healthcare system differences—including access to rehabilitation or institutional care—can further complicate cross-study comparisons on autonomy and cognitive outcomes.

Another factor contributing to heterogeneity across studies is the wide range of cognitive assessment tools employed, including the Montreal Cognitive Assessment (MoCA), Mini Mental State Examination (MMSE), and Mattis Dementia Rating Scale. Each instrument differs in its sensitivity to subtle cognitive changes, which may partly explain inconsistencies in reported outcomes.

Beyond the patient's individual trajectory, it is also essential to consider the broader psychosocial impact of STN-DBS, particularly on caregivers. While motor and non-motor symptom relief often improves the patient's QoL, the literature on caregiver outcomes remains limited. Existing research has primarily focused on caregivers’ self-reported burden, with less attention paid to their perceptions of patient improvement.^119–121^ This represents a gap in understanding, as caregivers’ perspectives can provide valuable insights into how DBS affects functional autonomy, emotional regulation, and overall home dynamics.

### Limitations and future directions

The current evidence on DBS is constrained by several methodological shortcomings. Many studies are based on relatively small samples, which limits statistical power and generalizability. Attrition between pre- and post-surgical assessments is also frequent, raising the risk of selection bias since patients with poorer outcomes are often those most likely to discontinue participation. Although high attrition may partly reflect the long travel distances for patients treated in specialized centers, it nonetheless undermines the reliability of long-term evaluations. A further limitation is the absence of appropriate control groups in numerous investigations, which makes it difficult to disentangle the effects of DBS from disease progression or pharmacological therapy. In addition, inconsistent reporting of key outcomes—particularly the lack of descriptive statistics for quality of life, autonomy, and non-motor symptoms—precludes inclusion of several studies in meta-analyses and restricts the robustness of quantitative conclusions. Finally, heterogeneity across study designs adds complexity: while multicenter studies dominate the field, single-center reports^56,65^ provide limited external validity, and few trials systematically evaluate both motor and non-motor domains with and without medication and stimulation.

Future research should directly address these gaps. Larger, multicenter trials are needed to improve statistical power and generalizability, while strategies such as telemedicine follow-up and logistical support may help reduce patient attrition and strengthen long-term outcome assessment. Incorporating matched comparator groups or randomized designs would allow clearer attribution of observed effects specifically to DBS. The adoption of standardized protocols for data collection and reporting—including full descriptive statistics for motor and non-motor outcomes—would enhance comparability and facilitate meta-analytic synthesis. Finally, harmonized methodologies that include comprehensive assessments of motor and non-motor symptoms under different stimulation and medication conditions are essential to disentangle the specific contributions of DBS and refine its role within an integrated therapeutic strategy.

## Conclusion

STN-DBS is a highly effective intervention for PD, significantly improving motor symptoms, reducing medication dependence, and enhancing autonomy in daily living. However, long-term outcomes vary, influenced by age, sex, cognitive function, and psychosocial factors. While early improvements in QoL and ADLs are well-documented, disease progression and levodopa-resistant symptoms present challenges in maintaining long-term independence. To fully optimize DBS benefits, a multidisciplinary post-surgical approach, integrating physical rehabilitation, cognitive monitoring, and psychological support, is essential. By refining patient selection criteria and postoperative care, DBS can continue to serve as a cornerstone therapy in improving both motor function and overall well-being in PD patients.

## References

[1] Hirtz D Thurman DJ Gwinn-Hardy K , et al. How common are the “common” neurologic disorders? Neurology 2007 Jan 30; 68: 326–337. 10.1212/01.wnl.0000252807.38124.a3 17261678

[2] Rocca WA . The burden of Parkinson’s disease: a worldwide perspective. Lancet Neurol 2018 Nov; 17: 928–929. 10.1016/S1474-4422(18)30355-7 30287052

[3] Dorsey ER Elbaz A Nichols E , et al. Global, regional, and national burden of Parkinson’s disease, 1990–2016: a systematic analysis for the global burden of disease study 2016. Lancet Neurol 2018 Nov; 17: 939–953. 10.1016/S1474-4422(18)30295-3 PMC6191528 30287051

[4] Pringsheim T Jette N Frolkis A , et al. The prevalence of Parkinson’s disease: a systematic review and meta-analysis. Mov Disord 2014 Nov 28; 29: 1583–1590. 10.1002/mds.25945 24976103

[5] Pagano G Ferrara N Brooks DJ , et al. Age at onset and Parkinson disease phenotype. Neurology 2016 Apr 12; 86: 1400–1407. 10.1212/WNL.0000000000002461 PMC4831034 26865518

[6] Koerts J König M Tucha L , et al. Working capacity of patients with Parkinson’s disease – A systematic review. Parkinsonism Relat Disord 2016 Jun; 27: 9–24. 10.1016/j.parkreldis.2016.03.017 27029582

[7] Yamabe K Kuwabara H Liebert R , et al. The burden of Parkinson’s disease(pd) in Japan. Value Health 2016 Nov; 19: A435.

[8] Keränen T Kaakkola S Sotaniemi K , et al. Economic burden and quality of life impairment increase with severity of PD. Parkinsonism Relat Disord 2003 Jan; 9: 163–168. 10.1016/s1353-8020(02)00097-4 12573872

[9] Hagell P Nordling S Reimer J , et al. Resource use and costs in a Swedish cohort of patients with Parkinson’s disease. Mov Disord 2002 Nov 24; 17: 1213–1220. 10.1002/mds.10262 12465059

[10] Schrag A . What contributes to quality of life in patients with Parkinson’s disease? J Neurol Neurosurg Psychiatry 2000 Sep 1; 69: 308–312. 10.1136/jnnp.69.3.308 PMC1737100 10945804

[11] Culicetto L Formica C Lo Buono V , et al. Possible implications of managing alexithymia on quality of life in Parkinson’s disease: a systematic review. Parkinsons Dis [Internet] 2024 Jan 1 [cited 2024 Oct 26]; 2024: 5551796. Available from: https://onlinelibrary.wiley.com/doi/full/10.1155/2024/5551796. 10.1155/2024/5551796 PMC11371456 39228428

[12] Santos García D de Deus Fonticoba T Suárez Castro E , et al. Non-motor symptoms burden, mood, and gait problems are the most significant factors contributing to a poor quality of life in non-demented Parkinson’s disease patients: results from the COPPADIS study cohort. Parkinsonism Relat Disord 2019 Sep; 66: 151–157. 10.1016/j.parkreldis.2019.07.031 31409572

[13] Bloem BR Okun MS Klein C . Parkinson’s disease. Lancet 2021 Jun; 397: 2284–2303. 10.1016/S0140-6736(21)00218-X 33848468

[14] Lo Buono V Palmeri R De Salvo S , et al. Anxiety, depression, and quality of life in Parkinson’s disease: the implications of multidisciplinary treatment. Neural Regen Res 2021; 16: 587. 10.4103/1673-5374.293151 PMC7996016 32985492

[15] Opara JA Brola W Leonardi M , et al. Quality of life in Parkinson’s disease. J Med Life 2012 Dec 15; 5: 375–381. PMC3539848 23346238

[16] Guo HJ Sapra A . Instrumental Activity of Daily Living. 2025. 31985920

[17] Edemekong PF Bomgaars DL Sukumaran S , et al. Activities of Daily Living. 2025. 29261878

[18] Hopkins RO Suchyta MR Kamdar BB , et al. Instrumental activities of daily living after critical illness: a systematic review. Ann Am Thorac Soc 2017 Aug; 14: 1332–1343. 10.1513/AnnalsATS.201701-059SR PMC5566273 28463657

[19] Bogetofte H Alamyar A Blaabjerg M , et al. Levodopa therapy for Parkinson’s disease: history, current Status and perspectives. CNS Neurol Disord Drug Targets 2020 Dec 24; 19: 572–583. 10.2174/1871527319666200722153156 32703142

[20] Goetz CG Poewe W Rascol O , et al. *Movement* disorder society task force report on the hoehn and yahr staging scale: status and recommendations the *Movement* disorder society task force on rating scales for Parkinson’s disease. Mov Disord 2004 Sep 30; 19: 1020–1028. 10.1002/mds.20213 15372591

[21] PD MED Collaborative Group. Long-term effectiveness of dopamine agonists and monoamine oxidase B inhibitors compared with levodopa as initial treatment for Parkinson’s disease (PD MED): a large, open-label, pragmatic randomised trial. Lancet 2014 Sep; 384: 1196–1205. 10.1016/S0140-6736(14)60683-8 24928805

[22] LeWitt PA Fahn S . Levodopa therapy for Parkinson disease. Neurology 2016 Apr 5; 86: S3–S12. 10.1212/WNL.0000000000002509 27044648

[23] Goetz CG Poewe W Rascol O , et al. Evidence-based medical review update: pharmacological and surgical treatments of Parkinson’s disease: 2001 to 2004. Mov Disord 2005 May 7; 20: 523–539. 10.1002/mds.20464 15818599

[24] Limousin P Krack P Pollak P , et al. Electrical stimulation of the subthalamic nucleus in advanced Parkinson’s disease. N Engl J Med 1998 Oct 15; 339: 1105–1111. 10.1056/NEJM199810153391603 9770557

[25] Bratsos S Karponis D Saleh SN . Efficacy and safety of deep brain stimulation in the treatment of Parkinson’s disease: a systematic review and meta-analysis of randomized controlled trials. Cureus 2018 Oct 22; 10: e3474. 10.7759/cureus.3474 PMC6318091 30648026

[26] Tödt I Al-Fatly B Granert O , et al. The contribution of subthalamic nucleus deep brain stimulation to the improvement in motor functions and quality of life. Mov Disord 2022 Feb 3; 37: 291–301. 10.1002/mds.28952 35112384

[27] Lee PS Crammond DJ Richardson RM . Deep Brain Stimulation of the Subthalamic Nucleus and Globus Pallidus for Parkinson’s Disease. In 2018. p. 207–21. 10.1159/000481105 29332085

[28] Okun MS . Deep-Brain stimulation for Parkinson’s disease. N Engl J Med 2012 Oct 18; 367: 1529–1538. 10.1056/NEJMct1208070 23075179

[29] Mansouri A Taslimi S Badhiwala JH , et al. Deep brain stimulation for Parkinson’s disease: meta-analysis of results of randomized trials at varying lengths of follow-up. J Neurosurg 2018 Apr; 128: 1199–1213. 10.3171/2016.11.JNS16715 28665252

[30] Hacker ML Currie AD Molinari AL , et al. Subthalamic nucleus deep brain stimulation may reduce medication costs in early stage Parkinson’s disease. J Parkinsons Dis 2016 Mar 30; 6: 125–131. 10.3233/JPD-150712 PMC4927876 26967937

[31] Hacker M Cannard G Turchan M , et al. Early subthalamic nucleus deep brain stimulation in Parkinson’s disease reduces long-term medication costs. Clin Neurol Neurosurg 2021 Nov; 210: 106976. 10.1016/j.clineuro.2021.106976 PMC8608728 34666273

[32] Volkmann J Daniels C Witt K . Neuropsychiatric effects of subthalamic neurostimulation in Parkinson disease. Nat Rev Neurol 2010 Sep 3; 6: 487–498. 10.1038/nrneurol.2010.111 20680036

[33] Lozano AM Mahant N . Deep brain stimulation surgery for Parkinson’s disease: mechanisms and consequences. Parkinsonism Relat Disord 2004 May; 10: S49–S57. 10.1016/j.parkreldis.2003.12.006 15109587

[34] Weaver FM . Bilateral deep brain stimulation vs best medical therapy for patients with advanced Parkinson disease: a randomized controlled trial. JAMA 2009 Jan 7; 301: 63. 10.1001/jama.2008.929 PMC2814800 19126811

[35] Mahlknecht P Foltynie T Limousin P , et al. How does deep brain stimulation change the course of Parkinson’s disease? Mov Disord 2022 Aug 12; 37: 1581–1592. 10.1002/mds.29052 PMC9545904 35560443

[36] Geraedts VJ Feleus S Marinus J , et al. What predicts quality of life after subthalamic deep brain stimulation in Parkinson’s disease? A systematic review. Eur J Neurol 2020 Mar 24; 27: 419–428. 10.1111/ene.14147 31876047

[37] Bove F Mulas D Cavallieri F , et al. Long-term outcomes (15 years) after subthalamic nucleus deep brain stimulation in patients with Parkinson disease. Neurology 2021 Jul 20; 97: e254–e262. 10.1212/WNL.0000000000012246 34078713

[38] Lezcano E Gómez-Esteban JC Tijero B , et al. Long-term impact on quality of life of subthalamic nucleus stimulation in Parkinson’s disease. J Neurol 2016 May 10; 263: 895–905. 10.1007/s00415-016-8077-4 26964542

[39] Bjerknes S Toft M Brandt R , et al. Subthalamic nucleus stimulation in Parkinson’s disease: 5-year extension study of a randomized trial. Mov Disord Clin Pract 2022 Jan 18; 9: 48–59. 10.1002/mdc3.13348 PMC8721829 35005065

[40] Accolla E Caputo E Cogiamanian F , et al. Gender differences in patients with Parkinson’s disease treated with subthalamic deep brain stimulation. Mov Disord 2007 Jun 15; 22: 1150–1156. 10.1002/mds.21520 17469208

[41] Hariz GM Limousin P Zrinzo L , et al. Gender differences in quality of life following subthalamic stimulation for Parkinson’s disease. Acta Neurol Scand 2013 Oct; 128: 281–285. 10.1111/ane.12127 23550919

[42] Chandran S Krishnan S Rao R , et al. Gender influence on selection and outcome of deep brain stimulation for Parkinson’s disease. Ann Indian Acad Neurol 2014; 17: 66. 10.4103/0972-2327.128557 PMC3992773 24753663

[43] Page MJ McKenzie JE Bossuyt PM , et al. The PRISMA 2020 statement: an updated guideline for reporting systematic reviews. BMJ [Internet] 2021 Mar 29 [cited 2024 Oct 27]; 372, Available from: https://www.bmj.com/content/372/bmj.n71. 10.1136/bmj.n71 PMC8005924 33782057

[44] Aviles-Olmos I Kefalopoulou Z Tripoliti E , et al. Long-term outcome of subthalamic nucleus deep brain stimulation for Parkinson’s disease using an MRI-guided and MRI-verified approach. J Neurol Neurosurg Psychiatry 2014 Dec; 85: 1419–1425. 10.1136/jnnp-2013-306907 PMC4451170 24790212

[45] Bezdicek O Mana J Růžička F , et al. The instrumental activities of daily living in Parkinson’s disease patients treated by subthalamic deep brain stimulation. Front Aging Neurosci 2022 Jun 17; 14: 886491. 10.3389/fnagi.2022.886491 PMC9247575 35783142

[46] Castrioto A Debû B Cousin E , et al. Long-term independence and quality of life after subthalamic stimulation in Parkinson disease. Eur J Neurol 2022 Sep 24; 29: 2645–2653. 10.1111/ene.15436 PMC9543065 35666167

[47] Dafsari HS Reddy P Herchenbach C , et al. Beneficial effects of bilateral subthalamic stimulation on non-motor symptoms in Parkinson’s disease. Brain Stimul 2016 Jan; 9: 78–85. 10.1016/j.brs.2015.08.005 26385442

[48] Dafsari HS Petry-Schmelzer JN Ray-Chaudhuri K , et al. Non-motor outcomes of subthalamic stimulation in Parkinson’s disease depend on location of active contacts. Brain Stimul 2018 Jul; 11: 904–912. 10.1016/j.brs.2018.03.009 29655586

[49] Dafsari HS Reker P Stalinski L , et al. Quality of life outcome after subthalamic stimulation in Parkinson’s disease depends on age. Mov Disord 2018 Jan 18; 33: 99–107. 10.1002/mds.27222 29150860

[50] Dafsari HS Silverdale M Strack M , et al. Nonmotor symptoms evolution during 24 months of bilateral subthalamic stimulation in Parkinson’s disease. Mov Disord 2018 Mar 21; 33: 421–430. 10.1002/mds.27283 29465787

[51] Dafsari HS Weiß L Silverdale M , et al. Short-term quality of life after subthalamic stimulation depends on non-motor symptoms in Parkinson’s disease. Brain Stimul 2018 Jul; 11: 867–874. 10.1016/j.brs.2018.02.015 29655587

[52] Straits-Tröster K Fields JA Wilkinson SB , et al. Health-Related quality of life in Parkinson’s disease after pallidotomy and deep brain stimulation. Brain Cogn 2000 Apr; 42: 399–416. 10.1006/brcg.1999.1112 10753487

[53] Jost TA Nelson B Rylander J . Quantitative analysis of the Oculus rift S in controlled movement. Disabil Rehabil Assist Technol 2021 Aug 18; 16: 632–636. 10.1080/17483107.2019.1688398 31726896

[54] Gronchi-Perrin A Viollier S Ghika J , et al. Does subthalamic nucleus deep brain stimulation really improve quality of life in Parkinson’s disease? Mov Disord 2006 Sep 8; 21: 1465–1468. 10.1002/mds.20943 16763974

[55] Fraix V . Clinical and economic results of bilateral subthalamic nucleus stimulation in Parkinson’s disease. J Neurol Neurosurg Psychiatry 2006 Apr 1; 77: 443–449. 10.1136/jnnp.2005.077677 PMC2077508 16543519

[56] Gorecka-Mazur A Furgala A Krygowska-Wajs A , et al. Activities of daily living and their relationship to health-related quality of life in patients with Parkinson disease after subthalamic nucleus deep brain stimulation. World Neurosurg 2019 May; 125: e552–e562. 10.1016/j.wneu.2019.01.132 30716489

[57] Nakamura K Yamanaka Y Higuchi Y , et al. Improved self-perceived performance for continence problems in patients with Parkinson’s disease after deep brain stimulation. Neurol Clin Neurosci 2019 Mar 22; 7: 51–56.

[58] Nazzaro JM Pahwa R Lyons KE . The impact of bilateral subthalamic stimulation on non-motor symptoms of Parkinson’s disease. Parkinsonism Relat Disord 2011 Sep; 17: 606–609. 10.1016/j.parkreldis.2011.05.009 21669545

[59] Besse-Pinot E Pereira B Durif F , et al. Preoperative REM sleep behavior disorder and subthalamic nucleus deep brain stimulation outcome in Parkinson disease 1 year after surgery. Neurology 2021 Nov 16; 97: e1994–e2006. 10.1212/WNL.0000000000012862 PMC8672430 34667082

[60] Plaha P Javed S Agombar D , et al. Bilateral caudal zona incerta nucleus stimulation for essential tremor: outcome and quality of life. J Neurol Neurosurg Psychiatry 2011 Aug 1; 82: 899–904. 10.1136/jnnp.2010.222992 21285454

[61] Williams A Gill S Varma T , et al. Deep brain stimulation plus best medical therapy versus best medical therapy alone for advanced Parkinson’s disease (PD SURG trial): a randomised, open-label trial. Lancet Neurol 2010 Jun; 9: 581–591. 10.1016/S1474-4422(10)70093-4 PMC2874872 20434403

[62] Cai YZ Zheng Y Li W , et al. Long-term outcomes of subthalamic nucleus deep brain stimulation for Parkinson’s disease in Singapore. Ann Acad Med Singap 2024 Aug 20; 53: 481–489. 10.47102/annals-acadmedsg.2023374 39230316

[63] Liu F Lang L Yang Y , et al. Predictors to quality of life improvements after subthalamic stimulation in Parkinson’s disease. Acta Neurol Scand 2018 Dec 13; 134: 346–352. 10.1111/ane.13056 30451276

[64] Lyons KE Pahwa R . Diagnosis and initiation of treatment in Parkinson’s disease. International Journal of Neuroscience 2011 Sep 30; 121: 27–36. 10.3109/00207454.2011.620197 22035027

[65] Jiang C Wang J Chen T , et al. Short- and long-term efficacy and safety of deep-brain stimulation in Parkinson’s disease patients aged 75 years and older. Brain Sci 2022 Nov 20; 12: 1588. 10.3390/brainsci12111588 PMC9688478 36421912

[66] Jiang JL Chen SY Tsai ST , et al. Long-Term effects of subthalamic stimulation on motor symptoms and quality of life in patients with Parkinson’s disease. Healthcare 2023 Mar 22; 11: 920. 10.3390/healthcare11060920 PMC10048478 36981577

[67] Sharma VD Lyons KE Nazzaro JM , et al. Deep brain stimulation of the subthalamic nucleus in Parkinson’s disease patients over 75 years of age. J Neurol Sci 2019 Apr; 399: 57–60. 10.1016/j.jns.2019.02.019 30772762

[68] Pusswald G Wiesbauer P Pirker W , et al. Depression, quality of life, activities of daily living, and subjective memory after deep brain stimulation in Parkinson disease—A reliable change index analysis. Int J Geriatr Psychiatry 2019 Nov 6; 34: 1698–1705. 10.1002/gps.5184 PMC6852657 31368144

[69] Patel NK . MRI Directed bilateral stimulation of the subthalamic nucleus in patients with Parkinson’s disease. J Neurol Neurosurg Psychiatry 2003 Dec 1; 74: 1631–1637. 10.1136/jnnp.74.12.1631 PMC1757425 14638880

[70] Siderowf A Jennings D Connolly J , et al. Risk factors for Parkinson’s disease and impaired olfaction in relatives of patients with Parkinson’s disease. Mov Disord 2007 Nov 15; 22: 2249–2255. 10.1002/mds.21707 17876851

[71] Zahodne LB Okun MS Foote KD , et al. Greater improvement in quality of life following unilateral deep brain stimulation surgery in the globus pallidus as compared to the subthalamic nucleus. J Neurol 2009 Aug 12; 256: 1321–1329. 10.1007/s00415-009-5121-7 PMC3045861 19363633

[72] Østergaard K Aa. Sunde N . Evolution of Parkinson’s disease during 4 years of bilateral deep brain stimulation of the subthalamic nucleus. Mov Disord 2006 May; 21: 624–631. 10.1002/mds.20776 16283616

[73] Ferrara J Diamond A Hunter C , et al. Impact of STN-DBS on life and health satisfaction in patients with Parkinson’s disease. J Neurol Neurosurg Psychiatry 2010 Mar 1; 81: 315–319. 10.1136/jnnp.2009.184127 19726415

[74] Drapier S Raoul S Drapier D , et al. Only physical aspects of quality of life are significantly improved by bilateral subthalamic stimulation in Parkinson’s disease. J Neurol 2005 May 23; 252: 583–588. 10.1007/s00415-005-0704-4 15778909

[75] Diaz AP Freitas FC de Oliveira Thais ME , et al. Variables associated with physical health-related quality of life in Parkinson’s disease patients presenting for deep brain stimulation. Neurol Sci 2016 Nov 25; 37: 1831–1837. 10.1007/s10072-016-2681-z 27457654

[76] Siderowf A Jaggi JL Xie SX , et al. Long-term effects of bilateral subthalamic nucleus stimulation on health-related quality of life in advanced Parkinson’s disease. Mov Disord 2006 Jun 6; 21: 746–753. 10.1002/mds.20786 16463342

[77] Dafsari HS Ray-Chaudhuri K Ashkan K , et al. Beneficial effect of 24-month bilateral subthalamic stimulation on quality of sleep in Parkinson’s disease. J Neurol 2020 Jun 9; 267: 1830–1841. 10.1007/s00415-020-09743-1 PMC7293679 32152689

[78] Jost ST Konitsioti A Loehrer PA , et al. Non-motor effects of deep brain stimulation in Parkinson’s disease motor subtypes. Parkinsonism Relat Disord 2023 Apr; 109: 105318. 10.1016/j.parkreldis.2023.105318 36842866

[79] Dafsari HS Ray-Chaudhuri K Mahlstedt P , et al. Beneficial effects of bilateral subthalamic stimulation on alexithymia in Parkinson’s disease. Eur J Neurol 2019 Feb 21; 26: 222. 10.1111/ene.13773 30107062

[80] Jost ST Aloui S Evans J , et al. Neurostimulation for advanced Parkinson disease and quality of life at 5 years. JAMA Netw Open 2024 Jan 18; 7: e2352177. 10.1001/jamanetworkopen.2023.52177 PMC10797423 38236600

[81] Lyons KE Pahwa R . Long-term benefits in quality of life provided by bilateral subthalamic stimulation in patients with Parkinson disease. J Neurosurg 2005 Aug; 103: 252–255. 10.3171/jns.2005.103.2.0252 16175854

[82] Mandat T Tykocki T Koziara H , et al. Subthalamic deep brain stimulation for the treatment of Parkinson disease. Neurol Neurochir Pol 2011; 45: 32–36. 10.1016/s0028-3843(14)60057-8 21384291

[83] Limousin P Pollak P Benazzouz A , et al. Bilateral subthalamic nucleus stimulation for severe Parkinson’s disease. Mov Disord 1995 Sep 12; 10: 672–674. 10.1002/mds.870100523 8552123

[84] Kleiner-Fisman G Fisman DN Sime E , et al. Long-term follow up of bilateral deep brain stimulation of the subthalamic nucleus in patients with advanced Parkinson disease. J Neurosurg 2003 Sep; 99: 489–495. 10.3171/jns.2003.99.3.0489 12959435

[85] Krack P Batir A Van Blercom N , et al. Five-Year follow-up of bilateral stimulation of the subthalamic nucleus in advanced Parkinson’s disease. N Engl J Med 2003 Nov 13; 349: 1925–1934. 10.1056/NEJMoa035275 14614167

[86] Martínez-Martín P Valldeoriola F Tolosa E , et al. Bilateral subthalamic nucleus stimulation and quality of life in advanced Parkinson’s disease. Mov Disord 2002 Mar 7; 17: 372–377. 10.1002/mds.10044 11921126

[87] Martinez-Martin P Deuschl G . Effect of medical and surgical interventions on health-related quality of life in Parkinson’s disease. Mov Disord 2007 Apr 30; 22: 757–765. 10.1002/mds.21407 17343275

[88] Diamond A . The effect of deep brain stimulation on quality of life in movement disorders. J Neurol Neurosurg Psychiatry 2005 Sep 1; 76: 1188–1193. 10.1136/jnnp.2005.065334 PMC1739801 16107348

[89] Spottke EA Volkmann J Lorenz D , et al. Evaluation of healthcare utilization and health status of patients with Parkinson’s disease treated with deep brain stimulation of the subthalamic nucleus. J Neurol 2002 Jun 1; 249: 759–766. 10.1007/s00415-002-0711-7 12111311

[90] Martinez Marinho M Broseghini Barcelos L Hyczy de Siqueira Tosin M , et al. Effect of bilateral deep brain stimulation on the subthalamic nucleus on patients with Parkinson’s disease: an observational and non-blinded study. Interdisciplinary Neurosurgery 2022 Mar; 27: 101380.

[91] Volkmann J . Deep brain stimulation for the treatment of Parkinson’s disease. J Clin Neurophysiol 2004 Jan; 21: 6–17. 10.1097/00004691-200401000-00003 15097290

[92] Limousin P Foltynie T . Long-term outcomes of deep brain stimulation in Parkinson disease. Nat Rev Neurol 2019 Apr 18; 15: 234–242. 10.1038/s41582-019-0145-9 30778210

[93] Lagrange E Krack P Moro E , et al. Bilateral subthalamic nucleus stimulation improves health-related quality of life in PD. Neurology 2002 Dec 24; 59: 1976–1978. 10.1212/01.wnl.0000037486.82390.1c 12499496

[94] Witt K Daniels C Reiff J , et al. Neuropsychological and psychiatric changes after deep brain stimulation for Parkinson’s disease: a randomised, multicentre study. Lancet Neurol 2008 Jul; 7: 605–614. 10.1016/S1474-4422(08)70114-5 18538636

[95] Just H Ostergaard K . Health-related quality of life in patients with advanced Parkinson’s disease treated with deep brain stimulation of the subthalamic nuclei. Mov Disord 2002 May 11; 17: 539–545. 10.1002/mds.10111 12112204

[96] Tröster AI Fields JA Wilkinson S , et al. Effect of motor improvement on quality of life following subthalamic stimulation is mediated by changes in depressive symptomatology. Stereotact Funct Neurosurg 2003; 80: 43–47. 10.1159/000075159 14745208

[97] Gray A McNamara I Aziz T , et al. Quality of life outcomes following surgical treatment of Parkinson’s disease. Mov Disord 2002 Jan 25; 17: 68–75. 10.1002/mds.1259 11835441

[98] Yamamoto T Uchiyama T Asahina M , et al. Urinary symptoms are correlated with quality of life after deep brain stimulation in Parkinson’s disease. Brain Behav 2018 Dec 19; 8: e01164. 10.1002/brb3.1164 PMC6305927 30451394

[99] Combs HL Folley BS Berry DTR , et al. Cognition and depression following deep brain stimulation of the subthalamic nucleus and globus Pallidus pars internus in Parkinson’s disease: a meta-analysis. Neuropsychol Rev 2015 Dec 12; 25: 439–454. 10.1007/s11065-015-9302-0 26459361

[100] Cury RG Galhardoni R Fonoff ET , et al. Effects of deep brain stimulation on pain and other nonmotor symptoms in Parkinson disease. Neurology 2014 Oct 14; 83: 1403–1409. 10.1212/WNL.0000000000000887 25217059

[101] Iranzo A . Sleep symptoms and polysomnographic architecture in advanced Parkinson’s disease after chronic bilateral subthalamic stimulation. J Neurol Neurosurg Psychiatry 2002 May 1; 72: 661–664. 10.1136/jnnp.72.5.661 PMC1737853 11971059

[102] Arai E Arai M Uchiyama T , et al. Subthalamic deep brain stimulation can improve gastric emptying in Parkinson’s disease. Brain 2012 May 1; 135: 1478–1485. 10.1093/brain/aws086 22522940

[103] Herzog J Weiss PH Assmus A , et al. Improved sensory gating of urinary bladder afferents in Parkinson’s disease following subthalamic stimulation. Brain 2007 Dec 3; 131: 132–145. 10.1093/brain/awm254 17977862

[104] Stemper B Berić A Welsch G , et al. Deep brain stimulation improves orthostatic regulation of patients with Parkinson disease. Neurology 2006 Nov 28; 67: 1781–1785. 10.1212/01.wnl.0000244416.30605.f1 17130410

[105] Martinez-Martin P Rodriguez-Blazquez C Forjaz MJ , et al. Impact of pharmacotherapy on quality of life in patients with Parkinson’s disease. CNS Drugs 2015 May 13; 29: 397–413. 10.1007/s40263-015-0247-x 25968563

[106] Stoker V Krack P Tonder L , et al. Deep brain stimulation impact on social and occupational functioning in Parkinson’s disease with early motor complications. Mov Disord Clin Pract 2020 Aug 3; 7: 672–680. 10.1002/mdc3.13015 PMC7396868 32775513

[107] Thobois S Ardouin C Lhommee E , et al. Non-motor dopamine withdrawal syndrome after surgery for Parkinson’s disease: predictors and underlying mesolimbic denervation. Brain 2010 Apr 1; 133: 1111–1127. 10.1093/brain/awq032 20237128

[108] Le Jeune F Drapier D Bourguignon A , et al. Subthalamic nucleus stimulation in Parkinson disease induces apathy. Neurology 2009 Nov 24; 73: 1746–1751. 10.1212/WNL.0b013e3181c34b34 19933975

[109] Drapier D Drapier S Sauleau P , et al. Does subthalamic nucleus stimulation induce apathy in Parkinson’s disease? J Neurol 2006 Aug 10; 253: 1083–1091. 10.1007/s00415-006-0177-0 16607469

[110] Porat O Cohen OS Schwartz R , et al. Association of preoperative symptom profile with psychiatric symptoms following subthalamic nucleus stimulation in patients with Parkinson’s disease. J Neuropsychiatry Clin Neurosci 2009 Oct; 21: 398–405. 10.1176/jnp.2009.21.4.398 19996248

[111] Schuepbach WMM Rau J Knudsen K , et al. Neurostimulation for Parkinson’s disease with early motor complications. N Engl J Med 2013 Feb 14; 368: 610–622. 10.1056/NEJMoa1205158 23406026

[112] Castrioto A Kistner A Klinger H , et al. Psychostimulant effect of levodopa: reversing sensitisation is possible. J Neurol Neurosurg Psychiatry 2013 Jan; 84: 18–22. 10.1136/jnnp-2012-302444 22991345

[113] Pagonabarraga J Kulisevsky J Strafella AP , et al. Apathy in Parkinson’s disease: clinical features, neural substrates, diagnosis, and treatment. Lancet Neurol 2015 May; 14: 518–531. 10.1016/S1474-4422(15)00019-8 25895932

[114] Saint-Cyr JA . Neuropsychological consequences of chronic bilateral stimulation of the subthalamic nucleus in Parkinson’s disease. Brain 2000 Oct 1; 123: 2091–2108. 10.1093/brain/123.10.2091 11004126

[115] Kim R Yoo D Choi JH , et al. Sex differences in the short-term and long-term effects of subthalamic nucleus stimulation in Parkinson’s disease. Parkinsonism Relat Disord 2019 Nov; 68: 73–78. 10.1016/j.parkreldis.2019.09.027 31621625

[116] Hariz MI Rehncrona S Quinn NP , et al. Multicenter study on deep brain stimulation in Parkinson’s disease: an independent assessment of reported adverse events at 4 years. Mov Disord 2008 Feb 15; 23: 416–421. 10.1002/mds.21888 18067188

[117] Bushnell CD Reeves MJ Zhao X , et al. Sex differences in quality of life after ischemic stroke. Neurology 2014 Mar 18; 82: 922–931. 10.1212/WNL.0000000000000208 PMC4211921 24510493

[118] Kim YE Kim HJ Kim HJ , et al. Impulse control and related behaviors after bilateral subthalamic stimulation in patients with Parkinson’s disease. J Clin Neurosci 2013 Jul; 20: 964–969. 10.1016/j.jocn.2012.07.020 23712053

[119] Leiknes I Tysnes OB Aarsland D , et al. Caregiver distress associated with neuropsychiatric problems in patients with early Parkinson’s disease: the Norwegian ParkWest study. Acta Neurol Scand 2010 Dec; 122: 418–424. 10.1111/j.1600-0404.2010.01332.x 20175757

[120] Chahine LM Ahmed A Sun Z . Effects of STN DBS for Parkinson’s disease on restless legs syndrome and other sleep-related measures. Parkinsonism Relat Disord 2011 Mar; 17: 208–211. 10.1016/j.parkreldis.2010.11.017 21216651

[121] Aarsland D Bronnick K Ehrt U , et al. Neuropsychiatric symptoms in patients with Parkinson’s disease and dementia: frequency, profile and associated care giver stress. J Neurol Neurosurg Psychiatry 2007 Jan 1; 78: 36–42. 10.1136/jnnp.2005.083113 PMC2117797 16820421

